# Safety and immunomodulatory efficacy of heat-killed *Mycolicibacterium manresensis* as a novel paraprobiotic in swine

**DOI:** 10.3389/fvets.2025.1660156

**Published:** 2025-10-15

**Authors:** Carmen Álvarez-Delgado, Inés Ruedas-Torres, José M. Sánchez-Carvajal, Karola Fristiková, Macarena Rodríguez-Ruiz, Fernanda Larenas-Muñoz, José J. Cerón, Francisco J. Pallarés, Pere J. Cardona, Irene M. Rodríguez-Gómez, Librado Carrasco, Jaime Gómez-Laguna

**Affiliations:** ^1^Department of Anatomy and Comparative Pathology and Toxicology, Pathology and Immunology Group (UCO-PIG), UIC Zoonosis y Enfermedades Emergentes ENZOEM, University of Córdoba, International Excellence Agrifood Campus ‘CeiA3’, Córdoba, Spain; ^2^United Kingdom Health Security Agency (UKHSA Porton Down), Salisbury, United Kingdom; ^3^Departamento de Patología y Medicina Preventiva, Facultad de Ciencias Veterinarias, Universidad de Concepción, Chillán, Chile; ^4^Interdisciplinary Laboratory of Clinical Analysis (Interlab-UMU), Regional Campus of International Excellence “Campus Mare Notrum”, University of Murcia, Murcia, Spain; ^5^Northern Metropolitan Clinical Laboratory, Microbiology Department, Hospital Universitari Germans Trias i Pujol, Badalona, Catalonia, Spain; ^6^Genetics and Microbiology Department, Universitat Autònoma de Barcelona, Barcelona, Catalonia, Spain

**Keywords:** piglet, nutritional alternative, paraprobiotic, *Mycolicibacterium manresensis*, immunomodulation

## Abstract

Given the increasing global concern about antimicrobial resistance, it is necessary to identify nutritional alternatives to antibiotics and trace elements in the porcine industry. The present study was carried out to evaluate the safety and effect of the paraprobiotic heat-killed *Mycolicibacterium manresensis* (hkMm) in weaned piglets. For this purpose, twenty-four-week-old piglets, were assigned to four experimental groups, and the diet of three of them was supplemented with 10, 50 and 100 ppm of hkMm, respectively, during a period of 70 d. Animals were monitored throughout the experiment, and weight data, blood, serum and saliva samples were collected every 2 weeks. At the end of the study, tissue samples were collected for histopathology, histomorphometry, immunohistochemical, and gene expression analyses. Supplemented animals did not show any adverse effects neither significant changes in their production parameters. Piglets supplemented with higher doses of hkMm exhibited a significant increase in salivary adenosine deaminase levels (*p* = 0.0042), along with a significant decrease in serum haptoglobin concentration (*p* = 0.0263). HkMm also appeared to induce a mild increase in circulating leukocyte populations at the end of the study, primarily due to elevated neutrophil counts, with smaller increases in lymphocytes and monocytes. Additionally, treated animals showed an increase in the number of regulatory T cells (FOXP3⁺) by immunohistochemistry, along with an increased *IFNG* response by RT-qPCR. Flow cytometry analysis in PBMC showed a decrease in the frequency of CD8β^+^ T cells (*p* = 0.0021), together with a higher number of γδ^+^ T cells in the treated animals throughout the study (*p* = 0.0327). On the other hand, histomorphometry analysis revealed a significant increase in mucosal height (*p* = 0.0195) and crypt depth (*p* = 0.0277) in the intestine of piglets receiving higher doses of hkMm. These results indicate that hkMm is a safe paraprobiotic with immunomodulatory potential, capable of enhancing intestinal integrity and absorptive capacity in weaned piglets, supporting its potential as a nutritional strategy to enhance gut health and reduce reliance on antimicrobial agents. Nevertheless, further studies in animals subjected to a challenge would be valuable to assess hkMm’s efficacy under immunological stress.

## 1 Introduction

There is no doubt that pigs rank as one of the most significant farm animals, both in terms of population and biomass, and it is important to highlight that the swine industry is one of the primary livestock sectors that utilize antimicrobials (1–4). The swine industry is a major global consumer of antimicrobials, driving the critical challenge of antimicrobial resistance (AMR). AMR poses a significant threat to both animal production and public health, as resistant pathogens and genes can disseminate through the food chain (1–5).

In the porcine industry, weaning is a particularly stressful phase, that in modern production systems, generally occurs 21–28 d after birth, often resulting in intestinal dysbiosis and alterations in gastrointestinal physiology, and immunology (6, 7). As a result, there is a decrease in the food intake, which in turn leads to a reduction in average daily weight gain. Furthermore, it increases vulnerability to digestive disorders due to changes in intestinal architecture and function (6). Moreover, psychosocial and dietary factors, such as separation from the mother, social stress and switching to solid feed, further compound the stress experienced during this stage (8–10).

One of the most frequent and economically significant consequences of this stress is the development of post-weaning diarrhea, a prevalent condition in pig farming worldwide. This illness leads to considerable morbidity, productivity losses, and increased mortality rates (11). Historically, pig farmers have been able to successfully wean pigs at an early age with minimal signs of gastrointestinal illness shortly after weaning, something that has largely been achieved using oral antimicrobials and zinc oxide (ZnO) (12).

In this regard, subtherapeutic doses of antimicrobials have been used for years as growth promoters in piglets. Although regulations have been implemented in some regions to restrict this practice (13), large quantities of antimicrobials continue to be used in several countries for the treatment and prevention of post-weaning diarrhea (6, 14). On the other hand, ZnO is a trace element that has long been used in high doses to also prevent diarrhea and control *Escherichia coli* F4 infections, as it positively influences both the absorption and structure of the small intestine, while also reducing intestinal permeability (15, 16). However, the absorption rate of zinc from dietary ZnO in pigs is relatively low, often cited around 20%, especially in weaned pigs, which enterocytes are fully developed to absorb it, in comparison with older pigs (17, 18). Moreover, it is a heavy metal with high concentration capacity in manure, which means that it can accumulate in soil and water for a long time (19). In addition, some authors have shown that high doses of this compound administered for periods longer than the first 2 weeks post-weaning may promote an increase of antibiotic resistance by bacteria (2, 20). These are the reasons why, since June 2022, therapeutic doses of ZnO are banned in the European Union (21).

In light of current regulatory restrictions on the use of antibiotics and ZnO in pig production, the search for functional feed ingredients that support intestinal health and immune function has intensified. Various strategies have been explored, including the incorporation of prebiotics, probiotics, organic acids, essential oils, liquid feeding systems, among others (16, 22–24). Among these, paraprobiotics, a next-generation probiotic, have recently emerged as a promising class of functional compounds. They are described as inactivated or non-viable microbial cells or cell fractions that provide benefits to the host, offering advantages such as easier production, safety, transportation, and storage compared to traditional probiotics (24, 25).

One such paraprobiotic of particular interest is heat-killed *Mycolicibacterium manresensis* (hkMm), recognized as a novel food by the European Food Safety Authority (EFSA) (26). Previous studies in humans and murine models have demonstrated its immunomodulatory effects, suggesting its potential to influence immune responses beneficially (27–29). However, its potential application as a dietary supplement in livestock remains unexplored. Evaluating the effects of hkMm supplementation in pigs, especially during the critical post-weaning phase, may offer novel insights into its role in promoting growth, modulating the immune response, and supporting gut health, thus contributing to the development of sustainable feeding approaches in the post-antibiotic era.

## 2 Materials and methods

### 2.1 Animals and experimental design

The present experiment was carried out to assess the beneficial effects on growth performance, immune system, and intestinal health of supplementing the diet of weaned piglets with hkMm (Nyaditum resae Manremyc, Barcelona, Spain), following the European Union guidelines (30) and being approved by the ethics committee of the Junta de Andalucía (reference 07/06/2023/39).

For this purpose, 20 four-week-old, intact, piglets of both sexes were sex- and weight-blocked and randomly distributed into 4 experimental groups of 5 animals, following a randomized block design based on sex and body weight. After 6 d of acclimatization, the diet of the three treated groups was supplemented with 10, 50 and 100 ppm of hkMm, respectively, during a period of 70 d. The diet of the fourth group was not supplemented and remained as control. The selected doses of hkMm were established based on a previous study conducted in a murine model (29). Each experimental group was separated in a pen under controlled housing conditions at the Experimental Animal Service (University of Córdoba, Córdoba, Spain), preventing contact between animals of different experimental groups. During the entire experiment, both feed and water were provided *ad libitum*. Supplementary Tables 1,2 represent the diet and nutrient composition of the feed provided to all the experimental groups along the study.

**Figure 1:**
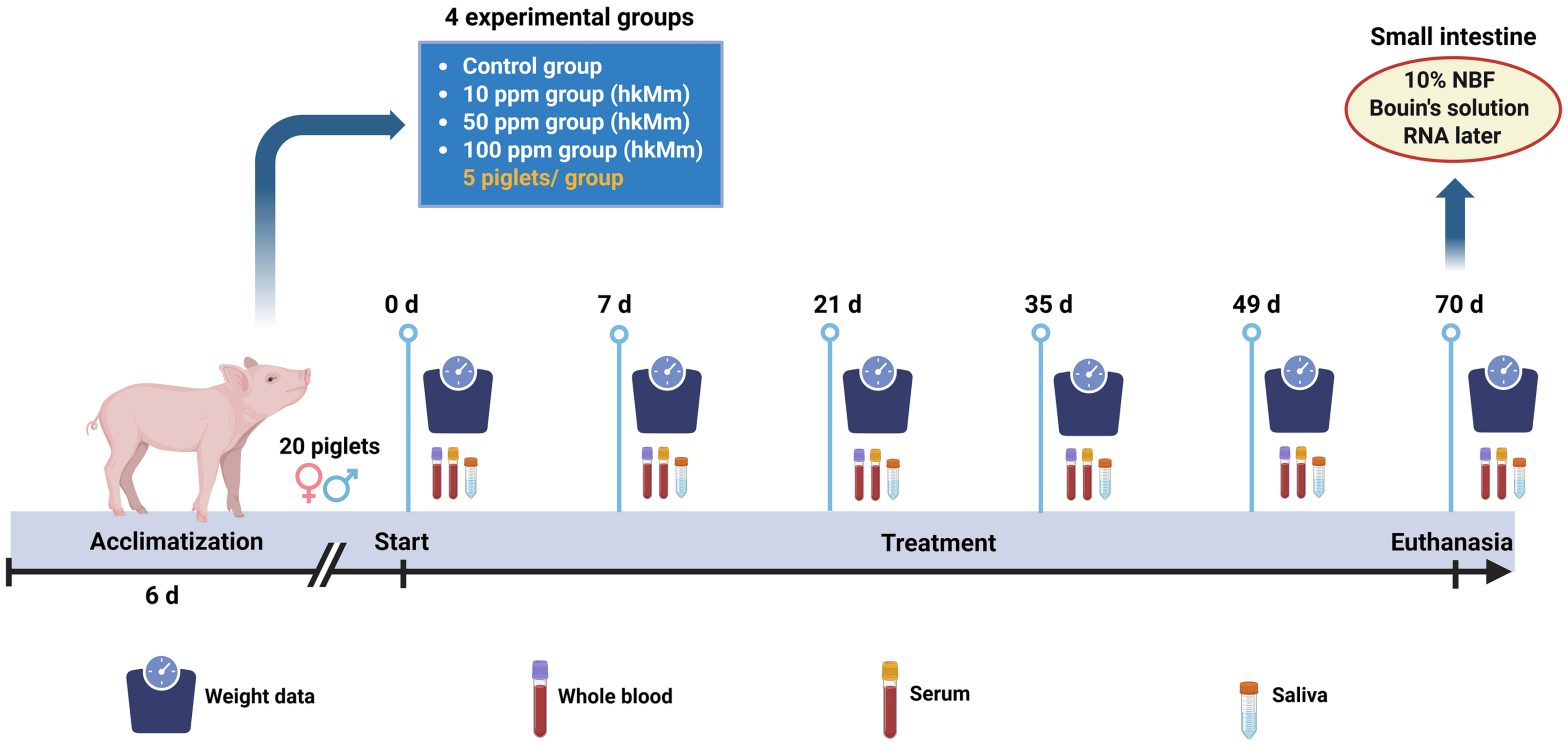
Experimental design and sampling representation throughout the study. Created with BioRender.com (31).

Animals were monitored throughout the experimental period, registering any behavioral and/or clinical sign, and at d 0, 7, 21, 35, 49, and 70 of the experiment, weight data was recorded and the average daily gain (ADG), and the feed conversion ratio (FCR) were calculated (Figure 1) (31). Moreover, as represented in Figure 1, samples of whole blood, and serum were collected by venipuncture from the vena cava, and saliva samples were taken with polypropylene sponges that were placed inside the animals’ mouth with forceps and thereafter placed inside a Salivette tube (Sarstedt, Nümbrecht, Germany).

At the end of the study, and as shown in Figure 1, all the animals were euthanized by intramuscular overdose of anesthetic (tiletamine-zolazepam 3 mg/kg, and medetomidine 0.05 mg/kg), and small intestine samples from duodenum, jejunum, and ileum were collected and fixed in 10% neutral buffered formalin (NBF) to perform histopathology and histomorphometry studies, and in 10% NBF and Bouin’s solution for immunohistochemical studies. Serial tissue samples from the same organs were collected in RNA stabilization solution (RNAlater Solution; Thermo Fisher Scientific, MA, USA) and stored at −80 °C to later perform RNA extraction. Additionally, samples from mesenteric lymph node (MLN) and Peyer’s patches were collected and embedded in cold Dulbecco’s Phosphate – Buffered Saline (DPBS, 1x, Corning, Manassas, VA, USA) to subsequently perform lymphocyte isolation, and phenotyping of lymphocyte subpopulations as well as activation markers.

### 2.2 Whole blood analysis

Whole blood samples were collected from the vena cava into commercial tubes (BD Vacutainer K2E EDTA 18.0 mg, UK) and analyzed via a differential white blood cell count at Laboratorio Veterinaria Garfia S.L. (Córdoba, Spain) to determine the number of lymphocytes, monocytes, neutrophils, eosinophils, and basophils. The results were reported as the number of cells per μL.

### 2.3 Saliva and sera analysis

Saliva samples were stored at room temperature (RT) throughout collection and transport to the laboratory, where they were centrifuged (3,220 × *g* at RT for 10 min). Sera samples were collected in commercial tubes (BD Vacutainer SST II Advance tubes, UK) and stored at RT until centrifugation at 2,465 × *g* at RT for 8 min. Supernatants from both saliva and sera were then collected in 1.5 mL tubes and stored at −80 °C until biochemical analyses were performed.

Immune system, inflammatory, and oxidative stress biomarkers [adenosine deaminase (ADA), haptoglobin (Hp), ferric reducing ability of plasma (FRAP), ferric reducing ability of saliva (FRAS), and cupric reducing antioxidant capacity (CUPRAC), respectively] were quantified in sera and saliva, except for Hp in the latter case, following different assays. ADA was measured in serum and saliva using a spectrophotometric assay commercially available (Adenosine Deaminase assay kit, Diazyme Laboratories, Poway, CA, USA), which has been previously validated for porcine (32). Salivary and serum CUPRAC were assayed by the method of Campos et al. (33), whereas FRAS and FRAP were measured by the method of Benzie and Strain (34). Hp was quantified in sera samples using a custom assay developed in-house that utilizes AlphaLISA technology (32).

### 2.4 Flow cytometry analysis

Flow cytometry analysis was performed on whole blood, MLN and Peyer’s patches samples to determine the proliferation of CD4^+^ and FoxP3^+^ T cells and the intracellular expression of IFN-γ and perforin within CD8β^+^ and γδ^+^ T cells.

#### 2.4.1 Isolation of peripheral blood mononuclear cells (PBMC) from whole blood

Whole blood samples were diluted 1:1 in DPBS and centrifuged (887 × *g* at RT for 30 min) through a density gradient (Lymphoprep, 1.077 g/mL) (Fisher Scientific, Vancouver, Canada). Then, PBMC located in the interphase were collected and washed twice in cold DPBS (470 × *g*, at 4 °C for 10 min), being the third and last wash performed under the same conditions but with culture medium [RPMI 1640 medium + 10% heat-inactivated Fetal Calf Serum (FCS) + 1% Penicillin/Streptomycin (P/S)].

#### 2.4.2 Isolation of MLN and Peyer’s patches cells

Lymphocyte isolation from MLN and Peyer’s patches was conducted in the same way. Briefly, tissue samples were placed in a Petri dish, kept moist with 1 mL cold DPBS and cut in small pieces (1–3 mm) with sterile scalpel blades. Tissue fragments were placed onto a 70-μm-pore-size filter wetted with DPBS and pressed using a syringe plunger. The filtered cell suspension was transferred to a 15 mL tube to perform two washes in cold DPBS (470 × *g* at 4 °C for 10 min). Then, cells were filtered through a mesh (1 mm × 1 mm) lined with a thin cotton layer. The mesh was rinsed with cold DPBS, and the resulting cell suspension was transferred to a 15 mL centrifuge tube to perform two additional washes, being the last one in culture medium.

#### 2.4.3 Intracellular cytokine assay

Freshly isolated PBMC and lymphocytes from MLN and Peyer’s patches were counted using Tuerk’s solution (Sigma-Aldrich, Missouri, USA). Subsequently, cells from each matrix and animal were seeded in replicates into round-bottom 96-well microtiter plates at a density of 500,000 cells per well in culture medium to analyze the production of IFN-γ and perforin in CD8β⁺ and γδ^+^ T cells. Cells were incubated for 18 h at 37 °C and 5% CO_2_. Four hours before harvesting, brefeldin A (BD GolgiPlug, BD Biosciences, Warsaw, Poland) was added to inhibit intracellular transport.

#### 2.4.4 Cell proliferation assay

For proliferation assay, freshly isolated PBMC and lymphocytes from MLN and Peyer’s patches were labeled with violet proliferation dye (CellTrace Violet Cell Proliferation Kit, Invitrogen, MA, USA) according to manufacturer’s recommendations. Briefly, cells from each matrix were adjusted to 20 × 10^6^ cells per mL, resuspended in 1 mL DPBS with 1 μL of violet dye stock solution, and incubated for 10 min in a water bath at 37 °C, after vigorous vortex. During incubation, samples were shaken several times. The labeling reaction was stopped by the addition of 2 mL FCS to each sample followed by incubation for 15 min at RT in darkness. After incubation, samples were resuspended and washed twice in culture medium (470 × *g* at RT for 10 min). Then, the supernatant was removed, and the cells were resuspended in 1 mL of culture medium to be quantified as above mentioned. After quantification, 500,000 cells per well were seeded into round-bottom 96-well microtiter plates as described above and incubated for 90 h. For proliferation analysis, the expression of CD4 and FoxP3 in combination with the activation markers CD25, and CD8α were analyzed.

#### 2.4.5 T cell population and cytokine profile analyses by flow cytometry

After either 18 or 90 h of incubation, plates were centrifuged (470 × *g* at RT for 5 min), and cells were harvested and stained for flow cytometric analysis as follows. Firstly, primary antibodies for surface markers of each T-cell subset were added and incubated for 15 min at RT (Tables 1, 2). Subsequently, cells were washed twice with 200 μL DPBS and fluorochrome-labelled isotype-specific secondary antibodies were incubated for 15 min at RT in darkness (Tables 1, 2). Thereafter, cells were washed twice with 200 μL of DPBS, and free binding sites of secondary antibodies were blocked with whole mouse IgG (Mouse IgG, whole molecule, ChromPure Jackson ImmunoResearch Laboratories, PA, USA).

**Table 1:** Primary, secondary, and intracellular antibodies used for T cell phenotyping in the intracellular cytokine staining (ICS).

Primary Ab	Clone	Isotype	Commercial brand
Swine CD8β	PG164A	IgG2a	Kingfisher Biotech
Swine TCR1-γδ	PGBL22A	IgG1	Kingfisher Biotech

Secondary Ab	Species Reactivity	Host	Fluorochrome	Commercial brand
IgG2a pAb	Mouse	Goat	Alexa Fluor 488	Invitrogen
IgG1 pAb	Mouse	Goat	Alexa Fluor 647	Invitrogen

Intracellular Ab	Clone	Host/Isotype	Conjugate	Commercial brand
IFN-γ mAb	P2G10	Mouse/IgG1, κ	PE	BD Pharmingen
Perforin mAb	δG9	Mouse/IgG2b, κ	PerCP-eFluor710	eBioscience

**Table 2:** Primary, secondary, and intracellular antibodies used in the proliferation analysis for CD4α^+^, CD25^+^, and CD8α^+^ T cells.

Primary Ab	Clone	Isotype	Commercial brand
CD4α mAb	MIL17	IgG2b	Bio-Rad Laboratories
CD25 mAb	K231.3B2	IgG1	Bio-Rad Laboratories
CD8α mAb	11/295/33	IgG2a	Bio-Rad Laboratories

Secondary Ab	Species Reactivity	Host	Fluorochrome	Commercial brand
IgG2b pAb	Mouse	Goat	Alexa Fluor 488	Invitrogen
IgG1 pAb	Mouse	Goat	Alexa Fluor 647	Invitrogen
IgG2a pAb	Mouse	Goat	PE/CY7	SouthernBiotech

Intracellular Ab	Clone	Host/Isotype	Conjugate	Commercial brand
FOXP3 mAb	FJK-16 s	Rat/IgG2a, κ	PE	eBioscience

Furthermore, for the intracellular cytokine staining (ICS), LIVE/DEAD Fixable Aqua Dead Cell Stain (Invitrogen) was used to discriminate dead cells. Then, cells were fixed and permeabilized using BD Cytofix/Cytoperm (BD Biosciences), and a cocktail of intracellular anti-IFN-γ and anti-perforin antibodies was used during 30 min at 4 °C in darkness to perform the intracellular staining (Table 1).

On the other hand, LIVE/DEAD Fixable Near-IR Dead Cell Stain (Invitrogen) was used for the proliferation panel to discriminate dead cells. Thereafter, cells were fixed and permeabilized with eBioscience Foxp3 (eBioscience), and, as shown in Table 2, intracellular staining was performed with the monoclonal antibody anti-FoxP3, following the same conditions as in the previous case for ICS.

Finally, samples were acquired in a flow cytometer (BD FACSCelesta SORP Flow Cytometer, BD Biosciences). Data were analyzed using FACSdiva (BD Biosciences) and FlowJo softwares.

### 2.5 Gross pathology, histological evaluation and histomorphometry analysis

At necropsy, post-mortem examination of the different organs was achieved by two pathologists and gross lesions, if any, were recorded. For the histopathological examination, four-micrometer tissue sections from duodenum, jejunum and ileum were stained with hematoxylin and eosin and blindly examined by two pathologists. The presence of any change at any level of the small intestine was evaluated and recorded.

Histomorphometric analysis was performed on the sections using the Aperio ImageScope software (v12.4.6.5003). Morphometry parameters, including mucosal height, villus width at two points, villus height on both sides, crypt depth, crypt width and the villus-to-crypt ratio were measured, as previously described (35). Additionally, to estimate the absorption surface of the intestinal mucosa, the surface amplification factor M was calculated, as described by Kisielinski et al. (36).

### 2.6 Immunohistochemical analysis

The Avidin–Biotin–Peroxidase Complex technique was performed to detect *in situ* the expression of FOXP3, CD3, and porcine IFNG in the samples from duodenum, jejunum, and ileum. Briefly, four-micrometer tissue sections from each sample, previously fixed in 10% NBF, for the analysis of FOXP3 and CD3, or in Bouin’s solution, for the evaluation of IFNG, were dewaxed and rehydrated in xylene and descending grades of alcohol. Then, the endogenous peroxidase activity was inhibited using 3% H_2_O_2_ solution in methanol for 30 min in darkness.

As indicated in Table 3, different pretreatments were carried out for FOXP3, CD3, and IFNG antigen retrieval. After phosphate-buffered saline (PBS) or tris buffered saline with 0.2% of tween 20 washes in the case of FoxP3, incubation with 100 μL of blocking solution was performed. Then, FOXP3, CD3, and IFNG primary antibodies were applied and incubated overnight (18 h) at 4 °C in a humidity chamber. For the negative controls, the primary antibody was replaced by either an isotype control or by blocking solution in each case to confirm the lack of non-specific biding. After incubation, slides were again incubated with the corresponding biotinylated secondary antibody for 30 min at RT in darkness. Then, the Avidin–Biotin–Peroxidase Complex (ABC Vector Elite, Vector Laboratories, Burlingame, CA, USA) was applied for 1 h under the same conditions. Labeling was visualized with the Vector NovaRED Substrate Kit, Peroxidase (HRP) (Vector Laboratories). Finally, slides were counter-stained with Harris hematoxylin, to later be dehydrated in ascending grades of alcohol and mounted with Eukitt mounting medium (Orsatec GmbH, Bobingen, Germany).

**Table 3:** Immunohistochemical methodology summary.

Antibody	Clone	Commercial brand	Dilution	Blocking solution	Antigen retrieval
FoxP3	FJK-16s	eBioscience	1:100	10% NGS	Citrate pH 6^a^
CD3	pAb	Dako	1:100	10% NGS	Protease^b^
IFN-γ	pAb	R&D Systems	1:20	2% BSA	Tween 20^c^

NGS, normal goat serum; BSA, bovine serum albumin.

a Citrate pH 6, autoclave treatment at 121 °C for 10 min.

b Protease from *Bacillus licheniformis* (Sigma-Aldrich, USA) for 8 min in agitation and in darkness.

c Nonionic detergent pretreatment (250 mL PBS + 500 μL Tween 20) for 10 min in agitation.

Labeled cells were identified and counted by using Olympus BX43 for FOXP3 and IFNG, or the QuPath software (version 0.3.2) for CD3, analyzing in each case 15 randomly villi at 20× magnification. The number of positive cells were determined in the villus epithelium, and in 3 different areas of the lamina propria: the villus, the upper half of the lamina propria with crypts and the lower half of the lamina propria with crypts. Supplementary Figure 1 shows the detailed analysis with QuPath software of the positive cells for the CD3 marker. Results were expressed as the median number of positive cells per 15 villi.

### 2.7 RT-qPCR analysis

#### 2.7.1 Total RNA extraction and cDNA synthesis

Total RNA was isolated from 50 mg of each small intestine section (duodenum, jejunum, and ileum), homogenized with 500 μL of NucleoZOL (Macherey-Nagel, Düren, Germany) using a portable homogenizer 150 (FisherBrand Thermo Fisher Scientific) and the NucleoSpin RNA Virus columns kit (Macherey-Nagel) according to manufacturer’s protocol. To remove genomic DNA, a DNase type I Ambion TURBO DNA-free kit (Invitrogen) was applied. Concentration and quality of the extracted RNA were determined by spectrophotometry using the Nanodrop 2000 spectrophotometer (Thermo Fisher Scientific). The iScript cDNA Synthesis Kit (Bio-Rad, CA, USA) was used to generate cDNA from total RNA as proposed by the manufacturer.

#### 2.7.2 Relative quantification by 2^−ΔΔCT^ method

The relative quantification of porcine genes of interest was carried out using the 2^−ΔΔCT^ method. The Cq values of target genes were normalized against the Cq values of selected reference genes (37). To identify the most stable reference genes, GeNorm analysis was conducted using qbase+ software (38) on a set of 8 candidate reference genes and 10 representatives cDNA samples. Four highly stable reference genes (0.5< average geNorm M ≤ 1) were identified as optimal for normalization. The normalization factor was determined using the arithmetic mean of the reference genes tyrosine 3-monooxygenase/tryptophan 5-monooxygenase activation protein zeta (*YWHAZ*), ribosomal protein L4 (*RPL4*), beta-2-microglobulin (*B2M*), and TATA box binding protein (*TBP*). The primer sequences for reference and target genes are provided in Table 4. Primers were designed using the Primer3Plus online tool^1^ (39), except as indicated.

**Table 4:** Primer sequences of the porcine reference genes (*YWHAZ*, *RPL4*, *B2M* and *TBP*) and the different target genes (*OCLN*, *TJP1*, *IL6*, *IL8*, *IL10*, *TNF*, *STAT1*, *TGFB1*, *FOXP3*, *CD3E*, *IFNG*, and *TRDC*).

Gene	Type	Sequences (5′ ➔ 3′)	Reference
*YWHAZ*	Reference gene	F: TGATGATAAGAAAGGGATTGTGG	(53)
		R: GTTCAGCAATGGCTTCATCA	
*RPL4*	Reference gene	F: GCGAAAAAGAGTCCCTCGGTTGT	Designed
		R: TCTCAGAACCGATTCAGGGACAC	
*B2M*	Reference gene	F: ACTTTTCACACCGCTCCAGT	(54)
		R: CGGATGGAACCCAGATACAT	
*TBP*	Reference gene	F: ACGTTCGGTTTAGGTTGCAG	(55)
		R: GCAGCACAGTACGAGCAACT	
*OCLN*	Target gene	F: CAGGTGCACCCTCCAGATTG	Designed
		R: TGGACTTTCAAGAGGCCTGGA	
*TJP1*	Target gene	F: ATCTCGGAAAAGTGCCAGGA	(56)
		R: CCCCTCAGAAACCCATACCA	
*IL6*	Target gene	F: AATCTGGGTTCAATCAGGAGACC	Designed
		R: ACTAATCTGCACAGCCTCGAC	
*IL8*	Target gene	F: AGGACCAGAGCCAGGAAGAG	Designed
		R: TGCCAGAACTGCAGCCTCA	
*IL10*	Target gene	F: TGAGAACAGCTGCATCCACTTC	(57)
		R: TCTGGTCCTTCGTTTGAAAGAAA	
*TNF*	Target gene	F: CGACTCAGTGCCGAGATCAA	(58)
		R: CCTGCCCAGATTCAGCAAAG	
*STAT1*	Target gene	F: CCTGTTGCGGTTCAGTGAGA	Designed
		R: GCATGGAAGTAAGGTTCGCCT	
*TGFB1*	Target gene	F: AGGGCTACCATGCCAATTTCT	(57)
		R: CCGGGTTGTGCTGGTTGT	
*FOXP3*	Target gene	F: CGCATGTTCGCCTTCTTCA	(59)
		R: AGGCTCAAGTTGTGGCGAAT	
*CD3E*	Target gene	F: CTGCCTCTTATCAGTTGGCG	Designed
		R: TTCAGGGCATGTCAGCTCTAC	
*IFNG*	Target gene	F: TGGTAGCTCTGGGAAACTGAATG	(57)
		R: GGCTTTGCGCTGGATCTG	
*TRDC*	Target gene	F: CACGTGACTTGGTATGGGGG	Designed
		R: TGGAATTAGGCTGACTTCGTGG	

The iTaq Universal SYBR Green Supermix kit (BioRad) was utilized according to the manufacturer’s protocol. Reactions were performed in duplicate with 45 ng of cDNA per sample and 0.5 μM of each primer, using the MyiQ 2 Two-Color Real-Time PCR Detection System (BioRad). The reaction conditions included polymerase activation at 95 °C for 20 s, followed by 40 cycles of denaturation at 95 °C for 15 s and annealing/extension at 60 °C for 30 s. A melting curve analysis (65–95 °C) was conducted to confirm the specificity of the amplicons. To ensure consistent quality of retro-transcription and detect inter-run variations, an inter-run calibrator sample with a known Cq value was included in each plate.

### 2.8 Statistical analysis

GraphPad Prism version 10.3.0 (217) for macOS (GraphPad Software, LLC) was employed for the generation of graphical representations and the performance of statistical analyses, using the D’Agostino and Pearson omnibus normality test. Since data did not follow a normal distribution, statistical differences were assessed by the Kruskal-Wallis non-parametric test for multiple comparisons followed by Dunn’s post-hoc test for multiple comparisons. Outliers were identified using the ROUT method (*Q* = 1%), which combines robust regression with false discovery rate control to objectively detect and remove outliers. *p*-value below 0.05 was considered indicative of statistical significance, indicated with *(*p* ≤ 0.05), **(*p* ≤ 0.01), and ***(*p* ≤ 0.001).

## 3 Results

### 3.1 Body weight, ADG and FCR

**Figure 2:**
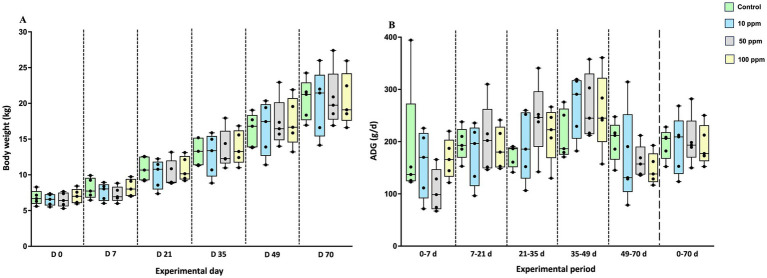
Graphical representation of body weight and Average Daily Gain (ADG) along the study. The panel includes **(A)** body weight measurements at different time points and **(B)** ADG calculated between consecutive time points. Data are represented as boxplots showing median, interquartile range (IQR), and minimum/maximum values. Black circles correspond to individual animal values within each group.

Body weight was homogeneous between experimental groups at the beginning of the study, with no statistically significant differences observed [median (IQR); Control group: 6.67 (2.69) kg, 10 ppm group: 6.58 (1.85) kg, 50 ppm group: 6.41 (2.37) kg, 100 ppm group: 7.00 (2.49) kg; *p* = 0.2608]. A progressive increase in body weight was observed throughout the study, as expected (Table 5; Figure 2).

**Table 5:** Data representation of performance parameters [body weight, average daily gain (ADG), and feed conversion ratio (FCR)] by experimental groups and dates.

Production parameters	Experimental date	Control	10 ppm	50 ppm	100 ppm
**Body weight (Kg)**	**d 0**	6.67 (IQR = 2.69)	6.58 (IQR = 1.85)	6.41 (IQR = 2.37)	7.00 (IQR = 2.49)
	**d 7**	7.73 (IQR = 3.46)	8.03 (IQR = 2.93)	6.93 (IQR = 2.84)	8.01 (IQR = 2.74)
	**d 21**	10.67 (IQR = 3.42)	10.78 (IQR = 4.88)	8.98 (IQR = 4.35)	10.15 (IQR = 3.94)
	**d 35**	13.29 (IQR = 3.90)	13.38 (IQR = 7.03)	12.27 (IQR = 6.97)	13.27 (IQR = 5.85)
	**d 49**	16.80 (IQR = 5.25)	17.45 (IQR = 8.95)	16.45 (IQR = 8.95)	16.65 (IQR = 8.70)
	**d 70**	21.25 (IQR = 7.30)	21.45 (IQR = 11.85)	19.75 (IQR = 10.50)	19.10 (IQR = 9.35)
**ADG (g/d)**	**Period 0–7 d**	137.1 (IQR = 271.4)	170.0 (IQR = 154.3)	98.6 (IQR = 98.6)	165.7 (IQR = 98.6)
	**Period 7–21 d**	192.9 (IQR = 85.0)	196.4 (IQR = 139.3)	202.1 (IQR = 163.6)	180.0 (IQR = 92.9)
	**Period 21–35 d**	186.4 (IQR = 49.3)	185.7 (IQR = 153.6)	246.4 (IQR = 198.6)	222.9 (IQR = 136.4)
	**Period 35–49 d**	186.4 (IQR = 105.0)	290.7 (IQR = 137.1)	245.0 (IQR = 145.7)	245.0 (IQR = 203.6)
	**Period 49–70 d**	211.9 (IQR = 102.4)	131.0 (IQR = 235.7)	157.1 (IQR = 76.2)	138.1 (IQR = 76.2)
	**Period 0–70 d**	206.3 (IQR = 75.7)	209.1 (IQR = 145.0)	193.3 (IQR = 132.0)	177.1 (IQR = 98.0)
**FCR (kg feed/kg body weight)**	**Period 0–21 d**	2.143	2.517	2.463	2.125
	**Period 0–35 d**	2.673	2.640	2.388	2.248
	**Period 0–49 d**	2.697	2.550	2.384	2.278
	**Period 0–70 d**	2.595	2.704	2.778	2.653

Each value represents the median and the Interquartile Range (IQR).

Moreover, although transient fluctuations in body weight and ADG were observed at specific time points, no statistically significant differences in ADG were found between groups (Table 5; Figure 2). Specifically, from day 0 to day 70, the ADG values were as follows [median (IQR): Control group: 206.3 (75.7) g/d, 10 ppm group: 209.1 (145.0) g/d, 50 ppm group: 193.3 (132.0) g/d, 100 ppm group: 177.1 (98.0) g/d; *p* = 0.9977].

Likewise, FCR remained similar between experimental groups throughout the study, with minor variations observed at certain time points (Table 5; Figure 2). For example, during the early phase (day 0–21), FCR was higher in the 10 ppm and 50 ppm groups (2.517 and 2.463, respectively) compared to the control (2.143) and 100 ppm group (2.125). Over time, FCR values gradually decreased in the 50 ppm and 100 ppm groups, reaching 2.384 and 2.278, respectively, by day 49. In contrast, by day 70, the 50 ppm and 10 ppm groups showed slightly elevated FCR values (2.778 and 2.704, respectively), while the control and 100 ppm groups remained lower (2.595 and 2.653, respectively).

### 3.2 White blood cell counting

**Figure 3:**
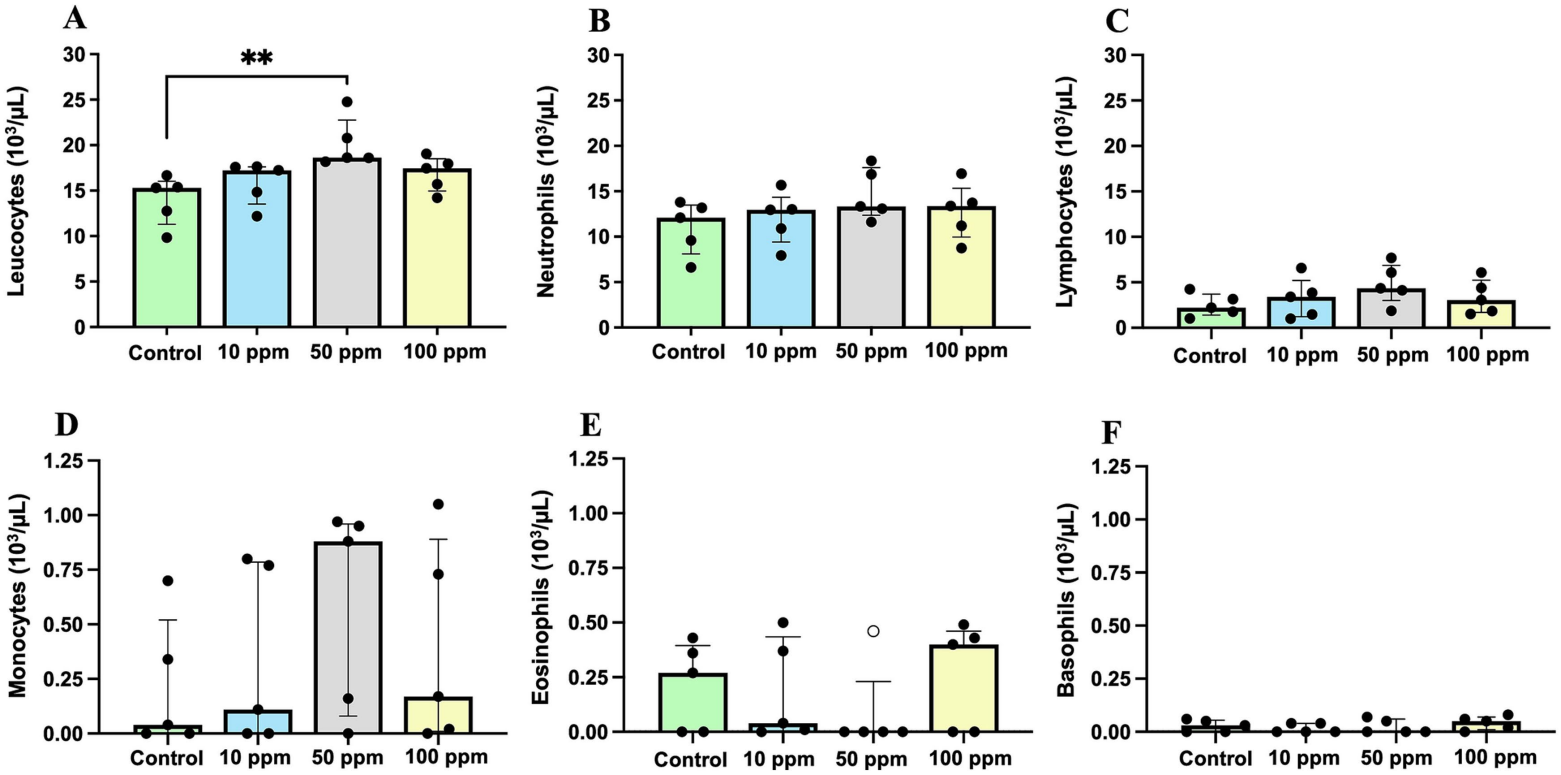
Graphical representation of leucocytes and the differential white blood cell counting at the end of the study (d 70). Panel includes **(A)** leucocytes, **(B)** neutrophils, **(C)** lymphocytes, **(D)** monocytes, **(E)** eosinophils, and **(F)** basophils. Statistical significance is indicated by *p* < 0.01 (**). Bars represent the median ± IQR. Black circles correspond to individual animal values within each group, and empty symbols represent outliers.

The most notable difference in white blood cell counts between experimental groups was observed at the end of the study (Figure 3). At this time point, total leukocyte count was significantly increased in the treated groups compared to the control group. This difference was statistically significant between the control and the 50 ppm group [15.31 (IQR = 6.84) ×10^3^/μL vs. 18.63 (IQR = 4.83) ×10^3^/μL, respectively; *p* = 0.0067] (Figure 3A). Similarly, neutrophil, lymphocyte, and monocyte counts were numerically higher in treated animals. Neutrophil, lymphocyte and monocyte counts were highest in the 50 ppm group; however, these increases were not statistically significant (Figures 3B–D).

### 3.3 Serum and saliva biomarkers

In serum, FRAP and CUPRAC values remained stable throughout the study and were highly similar across groups, with statistical differences being observed only on day 7 of the study for FRAP (Supplementary Figures 2A,B). On the other hand, in saliva, FRAS and CUPRAC values were lower compared to those observed in serum, and both antioxidant biomarkers showed a similar trend throughout the study and between the different experimental groups, showing a decrease as the study progressed (Supplementary Figures 2C,D).

**Figure 4:**
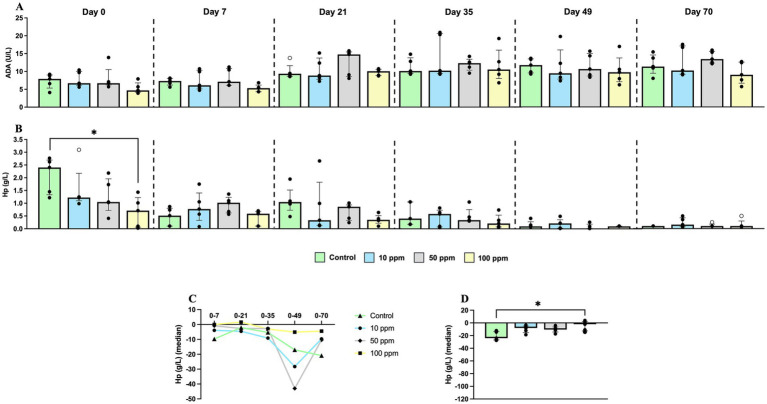
Graphical representation of serum biomarkers along the experimental study. Panel includes **(A)** adenosine deaminase (ADA), **(B)** haptoglobin (Hp), **(C)** Hp “fold-change” along the experiment, and **(D)** Hp “fold-change” at d 70. Statistical significance is indicated by *p* < 0.05 (*). Bars represent the median ± IQR. Black circles correspond to individual animal values within each group, and empty symbols represent outliers. In graph **(C)**, black triangles correspond to control values; black circles correspond to 10 ppm group values; black rhombuses correspond to 50 ppm group values; and black squares correspond to 100 ppm group values.

Regarding serum ADA values, despite temporary changes, no differences were observed among experimental groups along the study (Figure 4A). In relation to Hp, due to significant differences at the basal line on d 0 (Figure 4B), the kinetics for this parameter were evaluated as the evolution of the fold-change with respect to values at day 0. Accordingly, all experimental groups displayed a marked decrease in the serum Hp concentration which was significant for the control group when compared with 100 ppm group on day 70 (*p* = 0.0263) (Figure 4D). Noteworthy, at the end of the study, values became similar between all experimental groups as depicted in Figure 4B.

**Figure 5:**
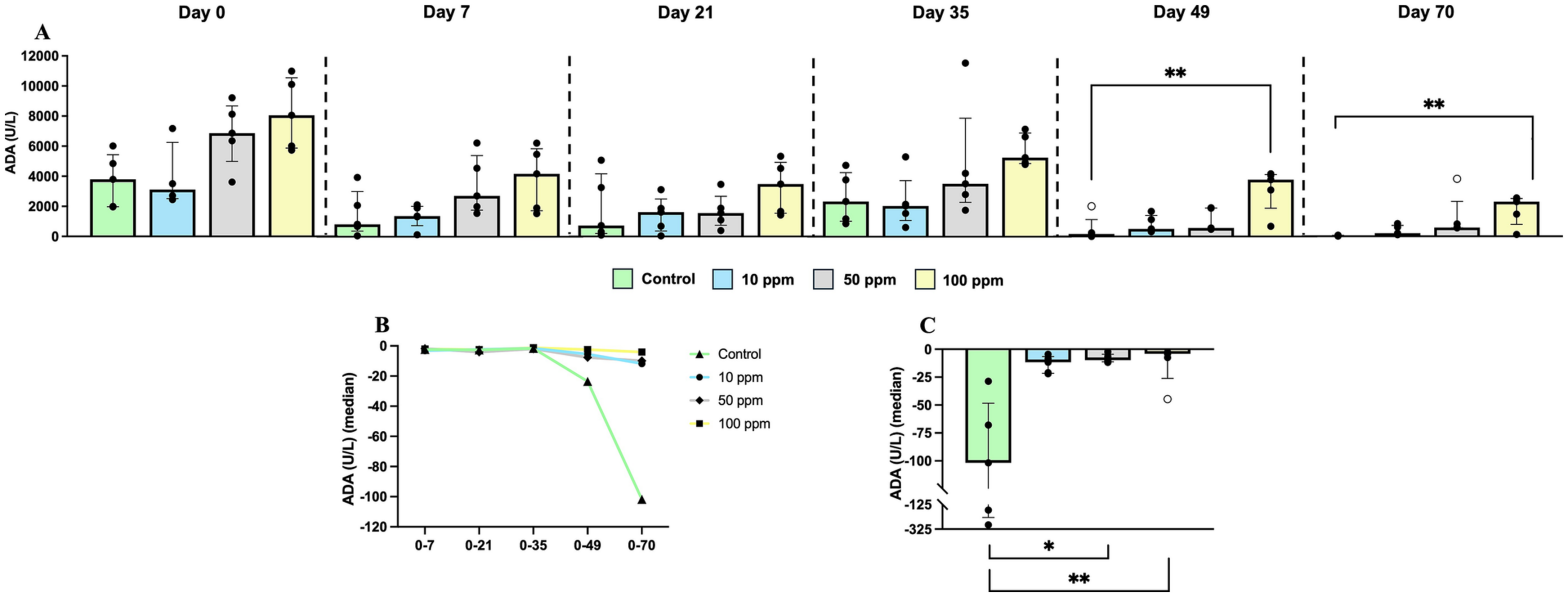
Graphical representation of ADA in saliva along the experimental study. Panel includes **(A)** ADA values along the experiment, **(B)** ADA “fold-change” along the experiment, and **(C)** ADA “fold-change” at d 70. Statistical significance is indicated by *p* < 0.05 (*), and *p* < 0.01 (**). Bars represent the median ± IQR. Black circles correspond to individual animal values within each group, and empty symbols represent outliers. In graph **(B)**, black triangles correspond to control values; black circles correspond to 10 ppm group values; black rhombuses correspond to 50 ppm group values; and black squares correspond to 100 ppm group values.

Regarding ADA values in saliva, much higher values of this enzyme than in serum were observed. This biomarker was consistently higher in the group supplemented with 100 ppm, and at lesser extent with 50 ppm of hkMm at all sampling points compared to the other experimental groups (Figure 5A). Nevertheless, the concentration of this enzyme decreased in all groups as the experiment progressed (Figure 5A). Statistically significant differences were observed at different time-points along the study, with all experimental groups presenting higher values than control group on day 49 and 70 (*p* = 0.0038, and *p* = 0.0052, respectively) (Figure 5A).

According to the unequal values of ADA in saliva at the beginning of the study, the fold-change for each experimental group along the study was calculated. Values of this enzyme remained stable along the study until day 35; from this time-point onwards there was a progressive decrease in the control group, which was only slightly displayed by experimental groups, with statistically significant differences on day 70 between control and supplemented animals (*p* = 0.0042) (Figures 5B,C).

### 3.4 Phenotypic characterization of T-lymphocyte subpopulations and activation markers

Flow cytometry analysis revealed noteworthy findings in lymphocyte subpopulations within PBMC; however, no remarkable differences were observed in MLN or Peyer’s patches (data not showed).

**Figure 6:**
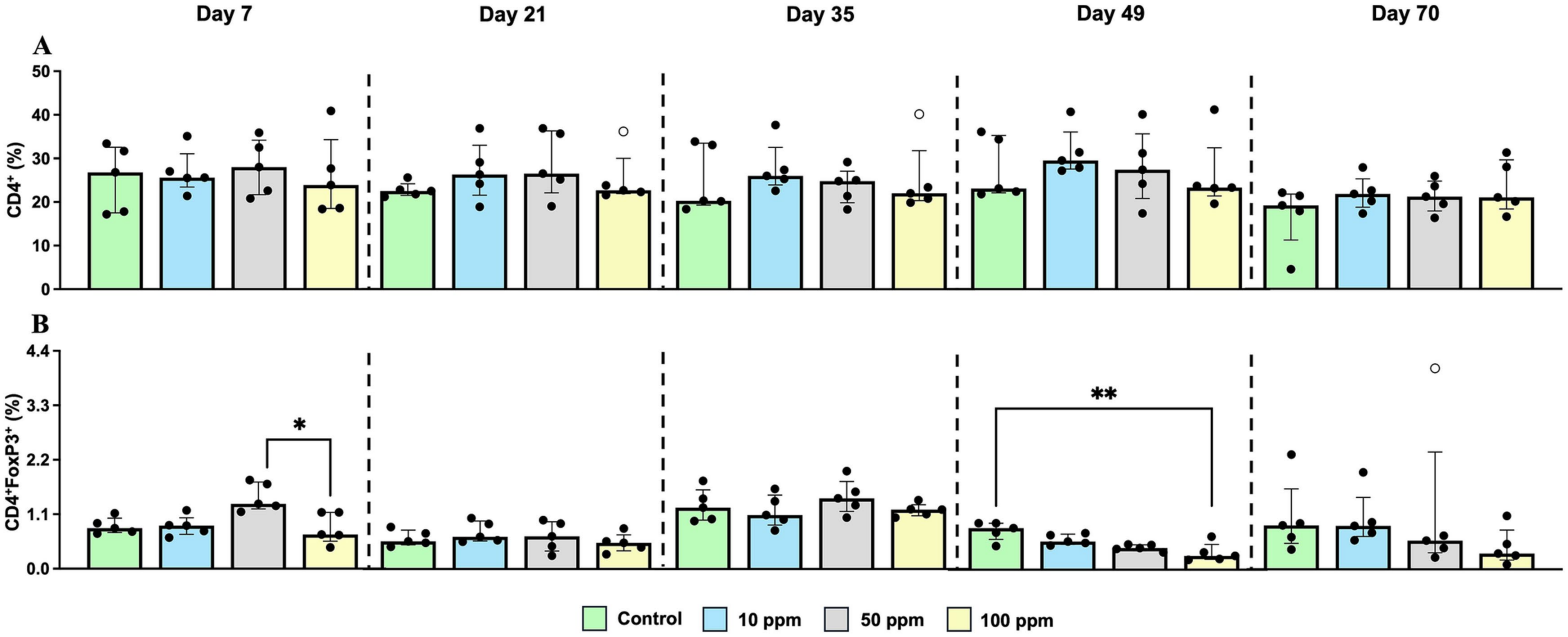
Graphical representation of the percentage of CD4^+^, and CD4^+^FoxP3^+^ T cells throughout the experiment in PBMC. Panel includes **(A)** CD4^+^ T cells (%), and **(B)** CD4^+^FoxP3^+^T cells (%). Statistical significance is indicated by *p* < 0.05 (*), and *p* < 0.01 (**). Bars represent median ± IQR. Black circles correspond to individual animal values within each group, and empty symbols represent outliers.

Phenotypic analysis of PMBC showed a transient increase of CD4^+^ cells in the treated groups (Figure 6A). Regarding CD4^+^FoxP3^+^ cells, the most notably change consisted of a decrease in the frequency of this subset, which was more marked according to increasing paraprobiotic doses, being statistically significant at d 49 of the study (*p* = 0.0094) (Figure 6B).

**Figure 7:**
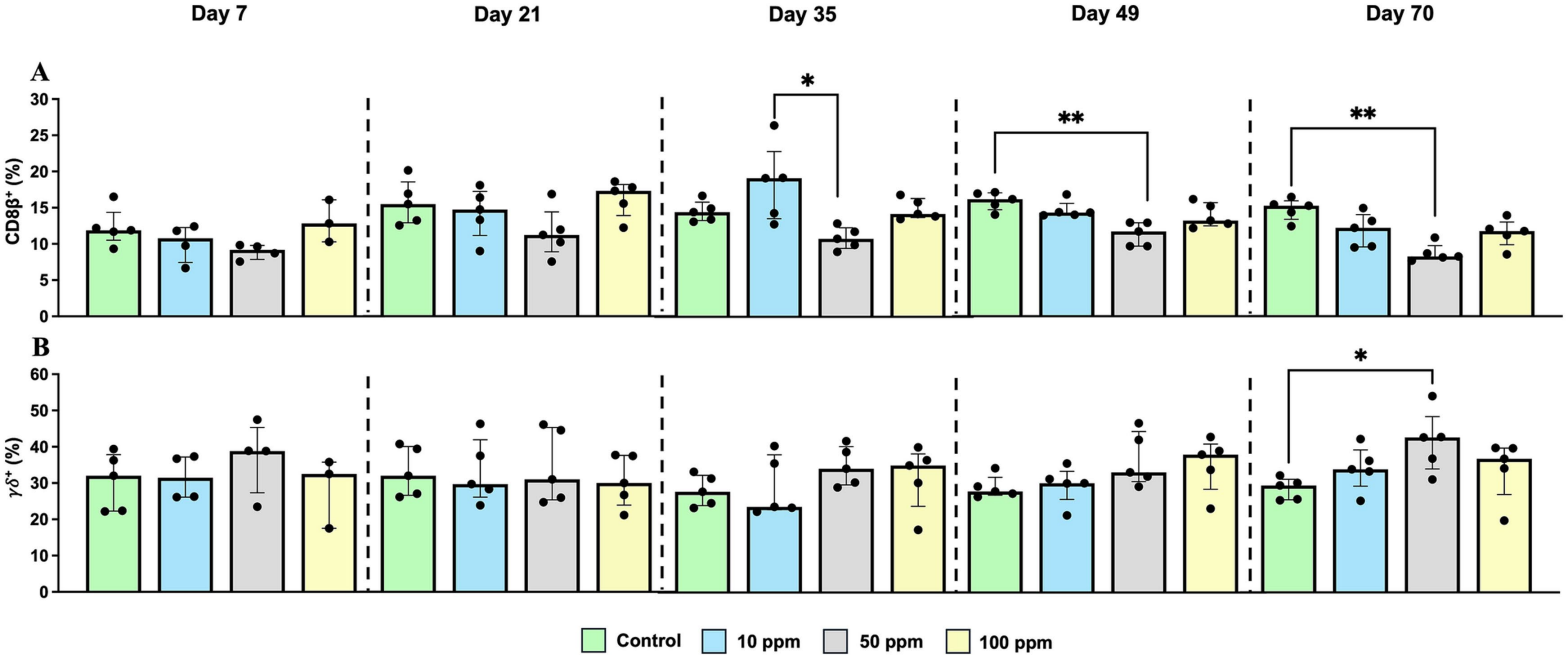
Graphical representation of the percentage of CD8β^+^, and γδ^+^ T cells throughout the experiment in PBMC. Panel includes **(A)** CD8β^+^ T cells (%), and **(B)** γδ^+^ T cells (%). Statistical significance is indicated by *p* < 0.05 (*), and *p* < 0.01 (**). Bars represent median ± IQR. Black circles correspond to individual animal values within each group.

CD8β^+^ cells consistently showed the lowest percentage in the 50 ppm group throughout the study, with a significant decrease with respect to control group from d 49 onwards (*p* = 0.0045 at d 49; *p* = 0.0021 at day 70) (Figure 7A). In contrast, γδ^+^ T cells exhibited the opposite trend, showing an increase in the PBMC of animals supplemented with hkMm as the experiment progressed, and by day 70, this increase became statistically significant between the control and the 50 ppm groups (*p* = 0.0327) (Figure 7B).

### 3.5 Histological evaluation and histomorphometric analysis

All animals in both the control and treated groups remained healthy throughout the study, and neither clinical signs nor macroscopic gastrointestinal lesions were detected in any of the animals. Moreover, no histopathological lesions were observed upon microscopic evaluation.

**Figure 8:**
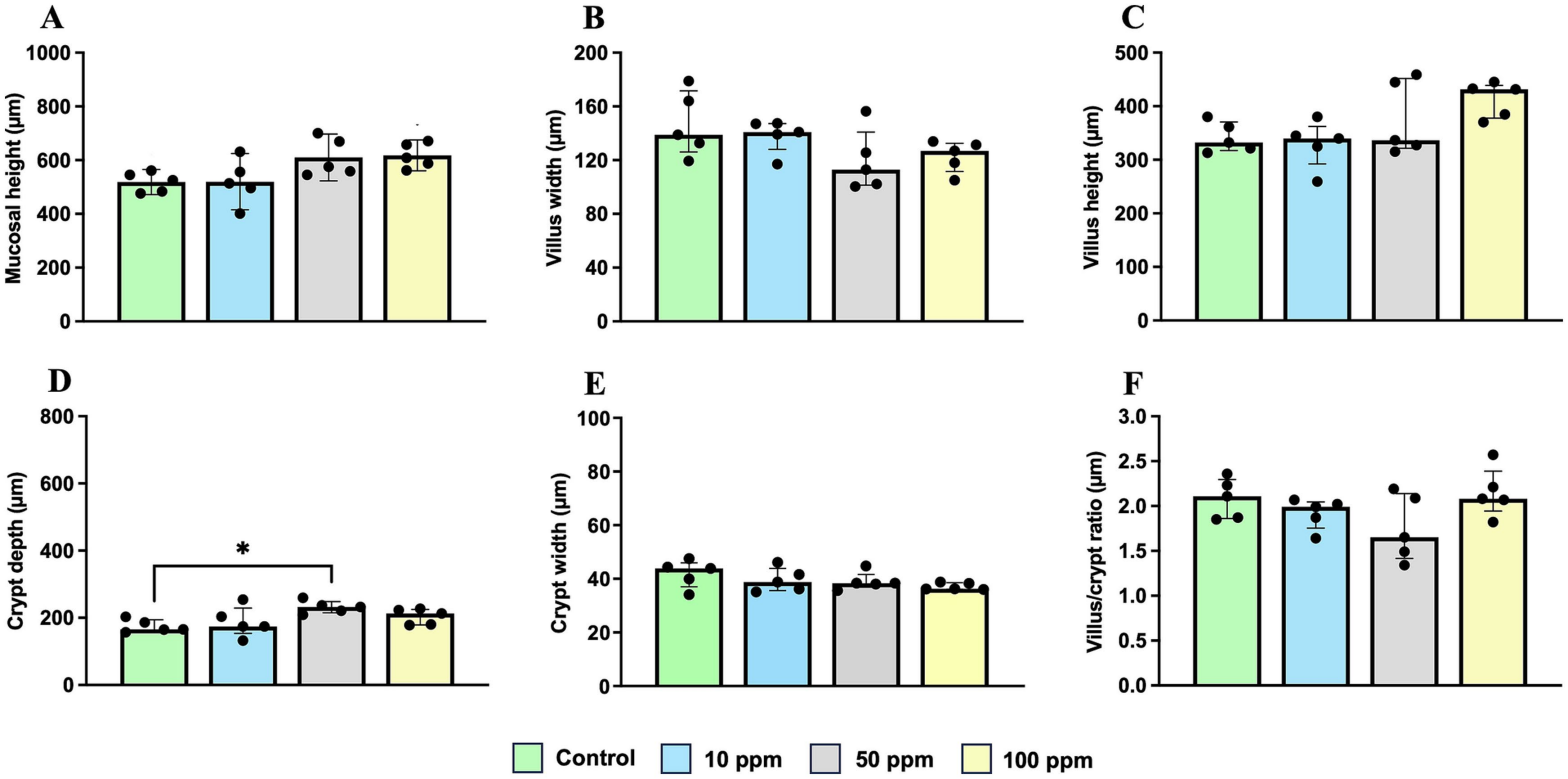
Graphical representation of the different measurements performed in the jejunum. The panel includes **(A)** mucosal height, **(B)** villus width, **(C)** villus height, **(D)** crypt depth, **(E)** crypt width, and **(F)** villus/crypt ratio. Statistical significance is indicated by *p* < 0.05 (*). Bars represent median ± IQR. Black circles correspond to individual animal values within each group.

Results of morphometric analysis are represented in Figure 8; Supplementary Figure 3. The most significant differences were observed at the jejunum level, although all three intestinal segments exhibit similar trends across the different histomorphometry parameters.

Mucosal height was greater in animals supplemented with hkMm, with statistically significant differences observed in the jejunum, where animals from the 100 ppm group showed higher values compared to the control group [608.4 (IQR = 109.3) and 525.5 (IQR = 85.4), respectively; *p* = 0.0195] (Figure 8A).

Villus height showed a similar upward trend, although without significant differences [431.5 (IQR = 74.9) and 332.2 (IQR = 66.9), respectively; *p* > 0.05], and this non-significant increase was also observed in the duodenum and ileum (Supplementary Figure 3).

Crypt depth followed the same pattern, with greater values in the jejunum and ileum of supplemented animals. In the jejunum, crypt depth was significantly increased in the 50 ppm group compared to the control one [232.0 (IQR = 50.7) and 165.6 (IQR = 45.7), respectively; *p* = 0.0277; Figure 8D].

Crypt width was generally similar between groups across all intestinal segments. However, a slight, non-significant decrease was observed in the jejunum of supplemented animals compared to controls (*p* > 0.05) (Figure 8E; Supplementary Figure 3).

On the other hand, as presented in Supplementary Figure 4, the M value was higher in the supplemented groups, particularly in the 50 ppm group at the level of the duodenum and jejunum, with the trend being more pronounced in the latter [5.77 (IQR = 3.12) in the control group vs. 6.30 (IQR = 1.91) in the 100 ppm group; Supplementary Figure 4B]. However, these differences were not statistically significant (*p* > 0.05).

### 3.6 Gene expression analysis

In the gene expression analysis, two animals could not be evaluated due to inadequate total RNA extraction: one belonging to the 50 ppm group (duodenum), and the other one to the control group (ileum). Considerable interindividual variability was observed for several target genes among animals within the same group in all the intestinal segments. Results are shown below for each group of target genes in the different intestinal segments.

#### 3.6.1 Tight junctions’ expression

As presented in Supplementary Figure 5, no relevant changes were observed in the expression of *TJP1* nor *OCLN* between experimental groups. However, a lower expression was evidenced in the treated groups, being more pronounced in the 50 ppm group at the level of the jejunum, and in the 100 ppm group at the level of the ileum, being statistically significant at this point, compared to the control group (*p* = 0.0051). In line with these results, a progressive trend toward lower expression of *OCLN* at the level of the ileum was detected, related to an increased paraprobiotic dose, being significant in the 100 ppm group, compared to the control one (*p* = 0.0044) (Supplementary Figure 5B).

#### 3.6.2 Cytokines expression

No significant changes were observed in the expression of proinflammatory cytokines (*IL8*, *IL6*, and *TNF*) between the experimental groups, not being observed an evident pattern according to each cytokine and evaluated intestinal segment (Supplementary Figures 6A–C).

Furthermore, no changes in the expression of the anti-inflammatory cytokine *IL10* were observed (Supplementary Figure 6D). Lastly, *IFNG* expression displayed an inverse behavior with respect to *STAT1* expression, the former displaying a trend to increase in treated animals as a function of increasing hkMm dose together with a proportional decrease in the expression of the latter (Supplementary Figures 6E,F).

#### 3.6.3 *CD3E*, *TRDC*, and *FOXP3* expression

As shown in Supplementary Figure 7A, *CD3E* showed a lower expression in the supplemented animals compared to the control group, which was significant at the level of the jejunum (*p* = 0.0221) and ileum (*p* = 0.0442), respectively, with respect to the control group. On the other hand, *TRDC* expression had a decreasing trend according to the progress of the intestinal segment (Supplementary Figure 7B), with significant changes in jejunum for 10 ppm and 50 ppm groups with respect to the control group. Finally, regarding *FOXP3*, there was a slight increase in expression at the level of the duodenum and ileum in the 50 ppm group (Supplementary Figure 7C).

### 3.7 Immunohistochemical analysis

#### 3.7.1 Evaluation of CD3^+^ immunolabelled cells

**Figure 9:**
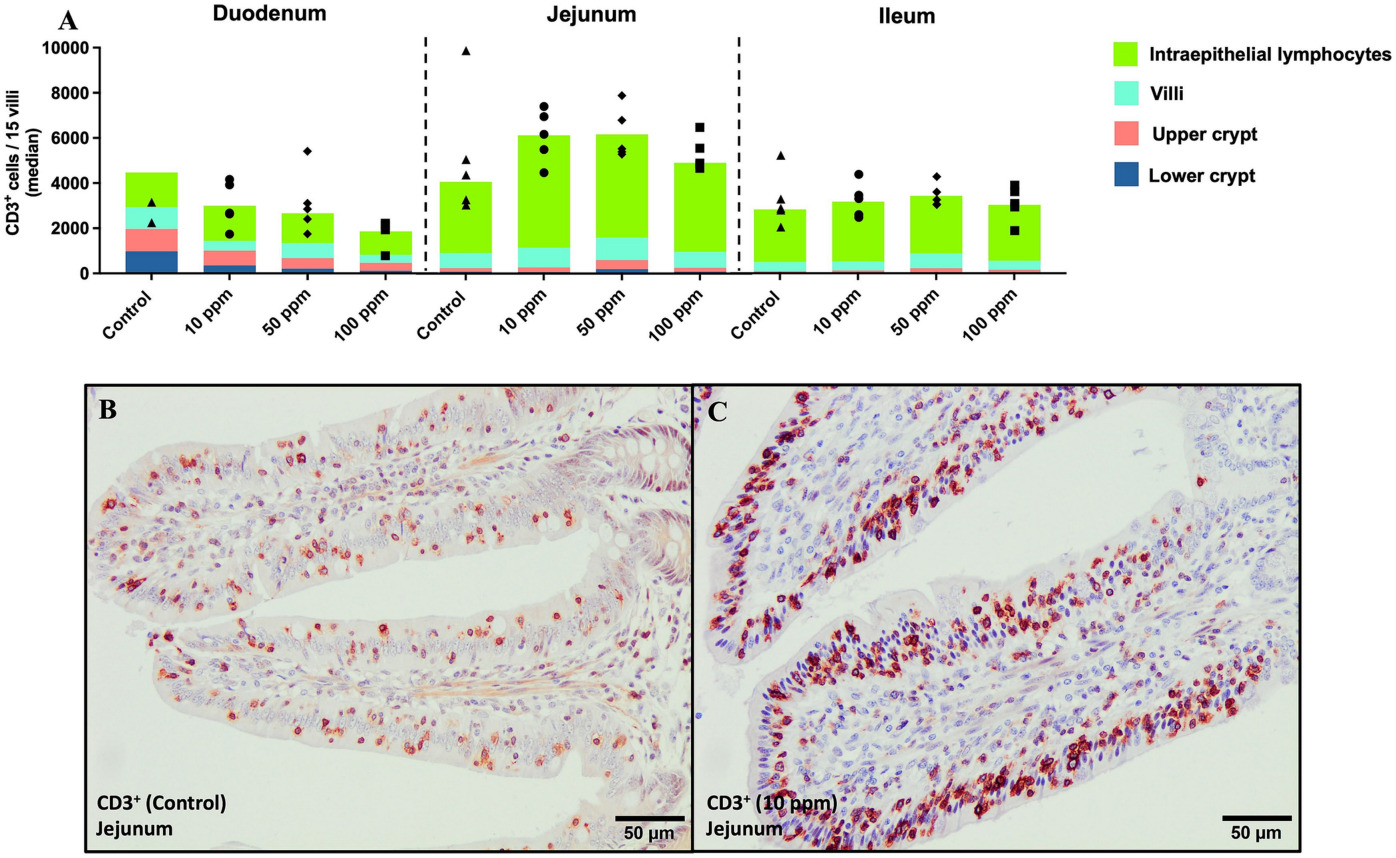
Graphical representation of CD3^+^ immunolabeling across the three segments of the small intestine at different tissue levels. Panel includes **(A)** CD3^+^ labeling in all intestinal segments and at different tissue levels per experimental group, **(B)** CD3^+^ cells at the level of the villi in jejunum in an animal from the control group, and **(C)** CD3^+^ cells at the level of the villi in jejunum in an animal from the 10 ppm group. Bars represent median ± IQR. Black triangles correspond to control individual values; black circles correspond to 10 ppm group individual values; black rhombuses correspond to 50 ppm group individual values; and black squares correspond to 100 ppm group individual values.

CD3 staining was detected in the membrane and cytoplasm of T lymphocytes (Figures 9B,C). The highest expression of CD3 was mainly observed in intraepithelial T lymphocytes across all three intestinal segments in all experimental groups, followed in a lesser extent by the lamina propria of villi, the upper crypts, and finally, the lower crypts (Figures 9B,C). In the jejunum and ileum, a higher expression of this marker was observed in the supplemented groups compared to the control one, being more pronounced in the jejunum as depicted in Figure 9A, with the highest number of positive cells being expressed in the 50 ppm group [5,468 (2,602), and 4,311 (6,841), respectively; *p* > 0.05], followed by the 10 ppm one.

#### 3.7.2 Evaluation of IFNG^+^ immunolabelled cells

**Figure 10:**
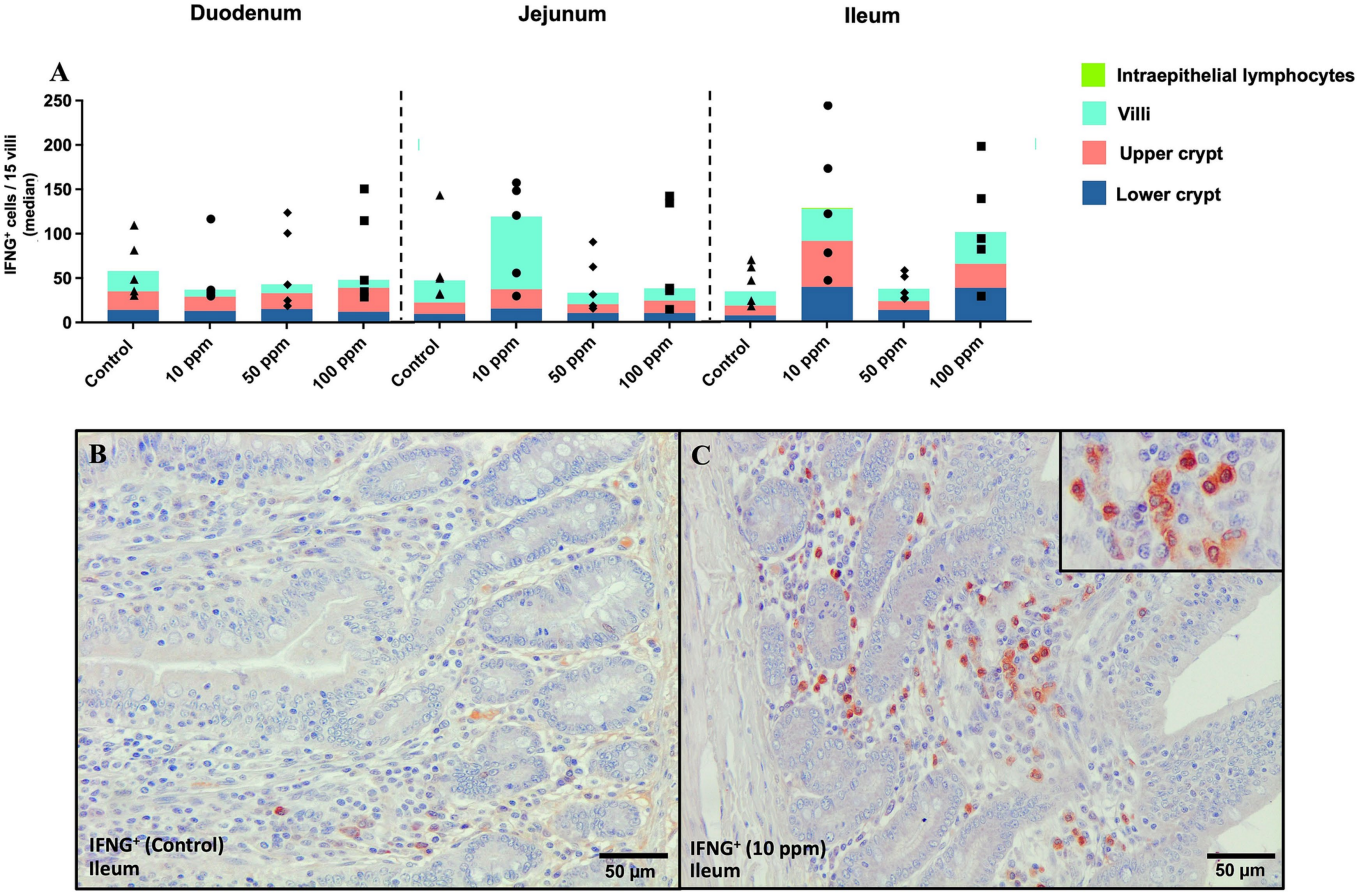
Graphical representation of IFNG^+^ immunolabeling across the three segments of the small intestine at different tissue levels. Panel includes **(A)** IFNG^+^ labeling in all intestinal segments and at different tissue levels per experimental group, **(B)** IFNG^+^ cells at the level of the upper crypts in ileum in an animal from the control group, and **(C)** IFNG^+^ cells at the level of the upper crypts in ileum in an animal from the 10 ppm group, inset shows a magnified view of the selected area. Bars represent median ± IQR. Black triangles correspond to control individual values; black circles correspond to 10 ppm group individual values; black rhombuses correspond to 50 ppm group individual values; and black squares correspond to 100 ppm group individual values.

IFNG staining was detected in the cytoplasm of lymphocyte-like cells (Figure 10C, inset), with expression varying across groups and tissue structures. Unlike previous marker, no clear expression pattern was observed in the different histological structures, as shown in Figure 10A. The most prominent differences were detected at the level of the jejunum and ileum, where the 10 ppm dose appeared to enhance the frequency of IFNG^+^ cells in both intestinal segments compared to the control animals [jejunum: 120 (128) vs. 49 (112), *p* > 0.05; ileum: 121 (197) vs. 46 (52), *p* > 0.05; Figure 10A]. Furthermore, the 100 ppm group also displayed a higher expression in the ileum compared to the remaining groups (Figure 10A). Whereas the increase of IFNG^+^ cells in the jejunum of 10 ppm animals was observed at the villi, this increase in the ileum of 10 ppm and 100 ppm was proportionally shared by villi and upper and lower crypt areas (Figure 10A). Figures 10B,C represent the differences in the immunolabeling at the level of the upper and lower crypts between a control animal and an animal treated with 10 ppm of the paraprobiotic, showing a higher number of positive cells in the latter.

#### 3.7.3 Evaluation of FOXP3^+^ immunolabelled cells

**Figure 11:**
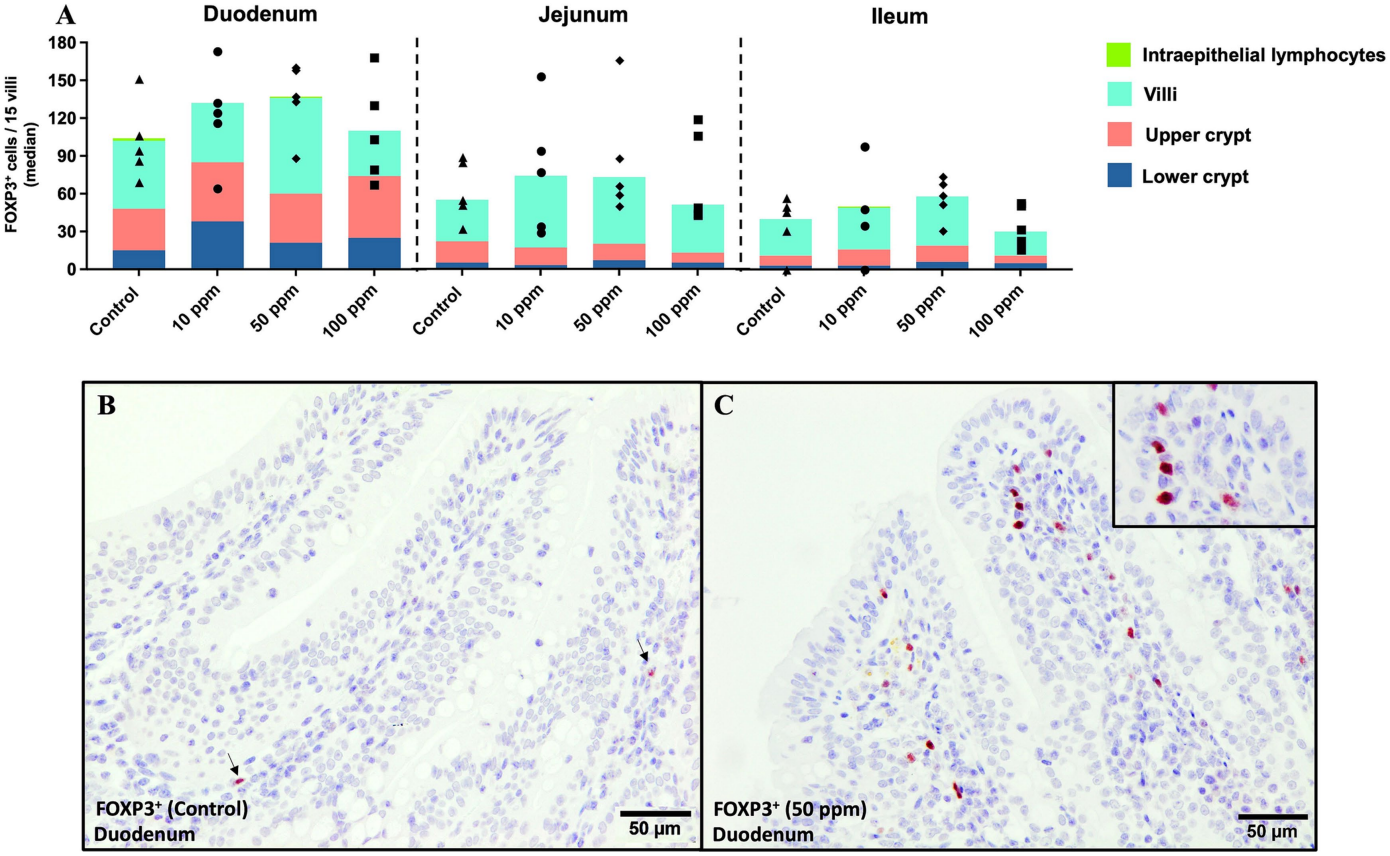
Graphical representation of FOXP3^+^ immunolabeling across the three segments of the small intestine at different tissue levels. Panel includes **(A)** FOXP3^+^ labeling in all intestinal segments, and at different tissue levels per experimental group, **(B)** FOXP3^+^ cells at the level of the villi in duodenum in an animal from the control group, and **(C)** FOXP3^+^ cells at the level of the villi in duodenum in an animal from the 50 ppm group, inset shows a magnified view of the selected area. Bars represent median ± IQR. Black triangles correspond to control individual values; black circles correspond to 10 ppm group individual values; black rhombuses correspond to 50 ppm group individual values; and black squares correspond to 100 ppm group individual values.

FOXP3 exhibited a nuclear labeling in lymphocyte-like cells (Figure 11C, inset). Overall, the highest expression of FOXP3 was observed in the lamina propria of the villi, followed by the upper and lower crypts, and, lastly, the epithelium (Figure 11A).

The results demonstrated an increased trend in the number of FOXP3^+^ cells in the treated groups compared to the control one, as shown in Figure 11A. The highest frequency of positive cells was observed in the duodenum, with the 50 ppm group showing the most pronounced labeling [136 (72) vs. 93 (82); *p* > 0.05; Figure 11A], mainly at the lamina propria of the villi as presented in Figures 11B,C. At the jejunum and ileum, the number of FOXP3^+^ cells followed a similar trend than in duodenum but with a lower number of positive cells (Figure 11A).

## 4 Discussion

In response to increasing restrictions on antimicrobial use in the swine industry, alternative nutritional strategies, such as the use of probiotics, prebiotics, organic acids, essential oils, and liquid feeding systems, among others (16, 20, 21, 24), are being explored to support animal health and performance, particularly in critical phases such as weaning (20, 40). Among these, next-generation probiotics such as the paraprobiotic hkMm, have shown immunomodulatory effects in both clinical and experimental models of tuberculosis (27–29), but their application in livestock species remains largely unexplored. The present study represents the first attempt to evaluate the safety and effect of hkMm supplementation in pigs, assessing its safety, immunological impact, and intestinal effects.

The absence of clinical signs, histopathological lesions, and changes in the production parameters throughout the study supports the safety of hkMm in weaned piglets, even at the highest doses tested. Moreover, the lack of significant upregulation of intestinal pro-inflammatory cytokines genes (*IL8, IL6, TNF*), and the downward trend in the serum concentration of Hp along the experiment in all groups suggest that hkMm does not trigger systemic or local inflammatory responses, since high serum levels of Hp have been associated with an inflammatory stimulus or an ongoing inflammatory process in pigs (41–43).

Beyond safety, several immune-related changes were observed. In this regard, in saliva, ADA values remained more stable in supplemented animals, particularly in those receiving the higher hkMm doses, which could be associated to a higher state of immune activation, being this enzyme a marker of adaptative cell-mediated immunity and lymphocyte T function (41). Additionally, ADA, especially the ADA2 isoform, plays a role in CD4⁺ T cell proliferation and in the differentiation of monocytes into macrophages (32, 44, 45), potentially explaining the increase in leukocyte populations observed at the end of the experiment, including lymphocytes, and monocytes.

Moreover, the apparent recruitment of regulatory T cells (FOXP3^+^) to the intestinal mucosa, particularly in the duodenum, together with their reduced frequency in PBMC, supports a possible local immunoregulatory effect (46). This observation is consistent with previous reports in humans and mice supplemented with hkMm, where an increase in regulatory T lymphocytes populations was associated with containment of tuberculosis-like lesions (28, 29). Although an increase in *IFNG* expression was also detected, which may seem contradictory with the rise in regulatory T cells, this dual activation pattern has been reported in balanced immune responses and may reflect a coordinated regulation (47). Further studies are needed to clarify the underlying mechanisms.

Histomorphometric analysis revealed significant increases in villus height, crypt depth and absorptive surface area, especially at the level of the jejunum in the animals supplemented with higher doses of the paraprobiotic. These structural improvements influence the overall height of the intestinal mucosa and have functional relevance. An increase in the villus height and absorption surface are associated with an enhance nutrient digestion and absorption, and contribute to faster growth (48–50). Similarly, crypts of Lieberkühn, which are invaginations of the intestinal epithelium that secrete digestive enzymes and mucus, are linked to better villi development and mucosal turnover (51). These results support that supplementation with hkMm could improve gut morphology, contributing to better digestion and intestinal absorption.

Flow cytometry analysis in PBMC showed a decrease in the frequency of CD8β^+^ T cells together with a higher number of γδ^+^ T cells in the treated animals throughout the study, particularly in those receiving the 50 ppm dose. These findings suggest a redistribution of immune cells, potentially involving recruitment of CD8β^+^ cells to the gut, along with the release of γδ^+^ T cells into the bloodstream, supported by the increased expression of CD3^+^ lymphocytes in the intestinal villi, mainly in the jejunum and ileum, as observed through immunohistochemistry. Similar changes in T cell subsets have been reported in a human clinical trial, where hkMm induced a rise in CD4⁺ T cells and non-classical monocytes (CD14^+^CD16^+^) (52). Furthermore, the increase in salivary ADA, already discussed above, also reflect this sift. Regrettably, no effective antibodies against CD8β and γδ T cells were available on our hands to assess both markers by immunohistochemical analysis.

Although this is a pilot study primarily designed to assess safety and dose response in the porcine model, the results obtained at the 50 ppm dose already indicate potentially beneficial effects on immune modulation and intestinal development. Therefore, this dose could serve as a promising starting point for future research. However, since no pathogenic challenge was applied in this study; further trials under infectious conditions would be essential to confirm whether the observed immune changes translate into enhanced protection or resilience against disease.

## 5 Conclusion

This study is the first to assess hkMm supplementation in pigs, indicating its safety and potential immunomodulatory benefits in the swine species. Observed changes indicated an increased leukocyte counts and γδ^+^ T cells in whole blood, enhanced ADA activity, and reduced serum Hp levels. In the intestine, a trend toward recruitment of regulatory T lymphocytes, along with an increase in IFNG expression was detected, pointing to a possible activation of the cell-mediated immunity pathways. These immunological changes were accompanied by signs of improved intestinal integrity and enhanced absorption capacity; however, direct casual relationships cannot be established based on the current data. Given these preliminary findings, future studies involving immunological or pathogenic challenges are warranted to better define the efficacy of hkMm supplementation under stress conditions and its potential impact on health and performance.

## Data Availability

The original contributions presented in the study are included in the article/Supplementary material, further inquiries can be directed to the corresponding author.

## Footnotes

^1^ www.primer3plus.com
