# Supplementary material for: Safety and immunomodulatory efficacy of heat-killed Mycolicibacterium manresensis as a novel paraprobiotic in swine
Source: Front Vet Sci. 2025 Oct 15;12:1660156. doi: 10.3389/fvets.2025.1660156 (PMC12570335; doi:10.3389/fvets.2025.1660156)
Supplement: Supplementary file 1 [file Data_Sheet_1.PDF]

Supplementary Material

1 Supplementary Tables and Figures

1.1 Supplementary Tables

Supplementary Table 1. Diet composition of the feed administered to the four experimental groups along the experimental study.

Raw Ingredients	Starter (g/kg)[1]	Adaptation (g/kg)[2]
Barley grain	320	403.07
Corn grain	316.87	224
Wheat grain	155.6	140
Soybean meal (47%)	120	141.33
Carob beans	-	30
Soybean hulls	-	30
Lard	15	10
Calcium carbonate	7.68	5.33
Dicalcium phosphate	8.86	5.72
Sodium chloride	4	4
Lysine HCl	-	2.55
Organic acids	2	-
Pre-Mix	50	4

[1]Starter diet was administered to all the animals up to 11 weeks of age.

[2]Adaptation diet was administered to the different experimental groups from 11 weeks of age to the end of the study.

Supplementary Table 2. Nutrient composition of the diet administered to the four experimental groups along the experimental study.

Nutrient composition (%)	Starter[1]	Adaptation[2]
Crude protein	15.40	15.40
Crude fat	3.70	3.07
Crude fiber	3.39	4.89
Crude ash	5.07	4.30
Calcium	0.70	0.56
Phosphorus	0.49	0.43
Lysine	1.10	0.88
Methionine	0.41	0.23
Sodium	0.23	0.17

[1]Starter diet was administered to all the animals up to 11 weeks of age.

[2]Adaptation diet was administered to the different experimental groups from 11 weeks of age to the end of the study.

1.2 Supplementary Figures

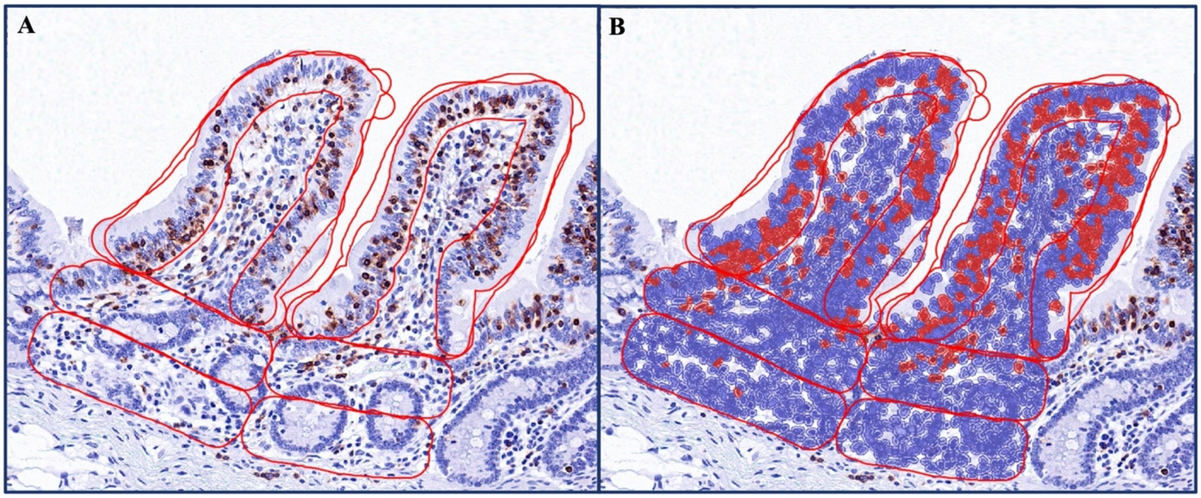

Supplementary Figure 1. Analysis of $CD3^+$ cells using QuPath software. Panel includes (**A**) Representative image showing selected histological structures used for cell counting, and (**B**) Visualization of cell classification, indicating CD3-positive (red), and CD3-negative (blue) cells.

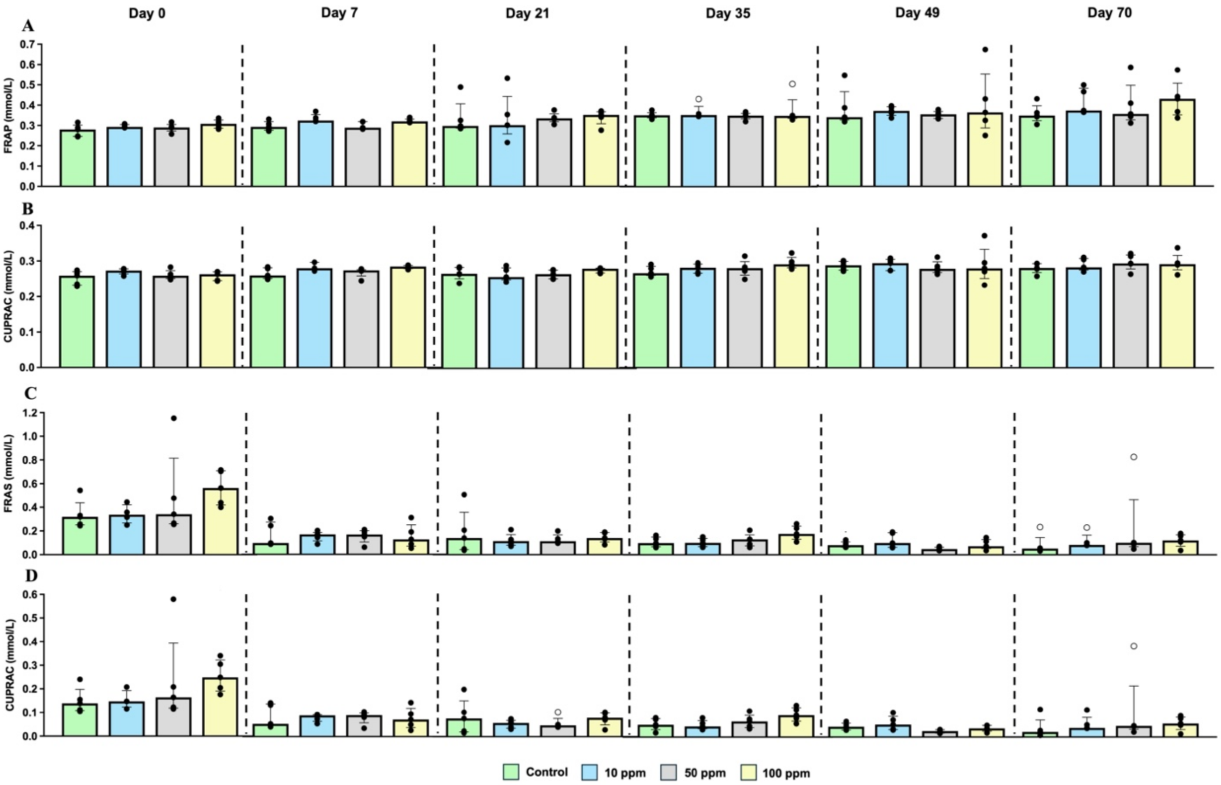

Supplementary Figure 2. Graphical representation of serum and saliva biomarkers along the experimental study. Panel includes (**A**) ferric reducing activity of plasma (FRAP), (**B**) cupric reducing antioxidant capacity (CUPRAC), (**C**) ferric reducing activity of saliva (FRAS), and (**D**) CUPRAC of saliva. Bars represent the median ± IQR. Black circles correspond to individual animal values within each group, and empty symbols represent outliers.

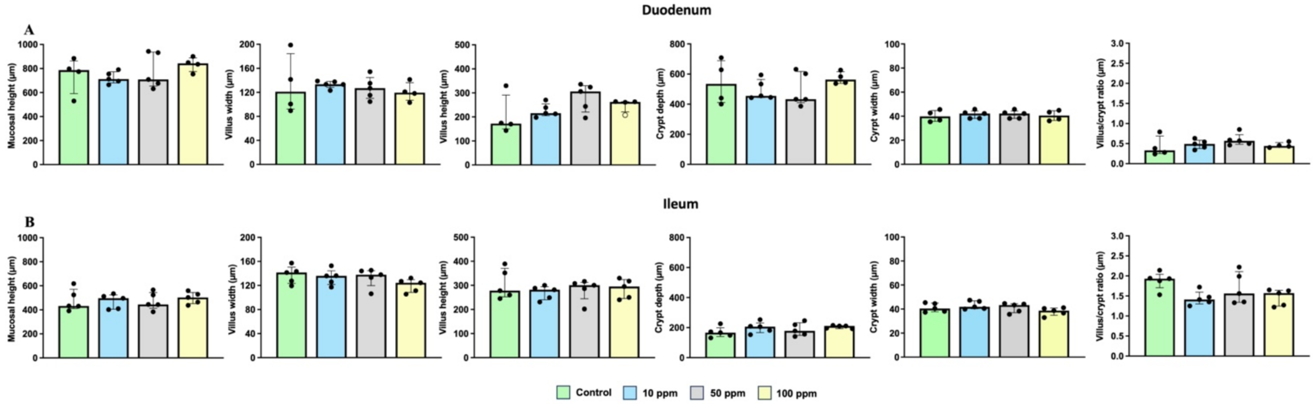

Supplementary Figure 3. Graphical representation of the different measurements performed in the (**A**) duodenum, and (**B**) ileum. Bars represent median ± IQR. Black circles correspond to individual animal values within each group, and empty symbols represent outliers.

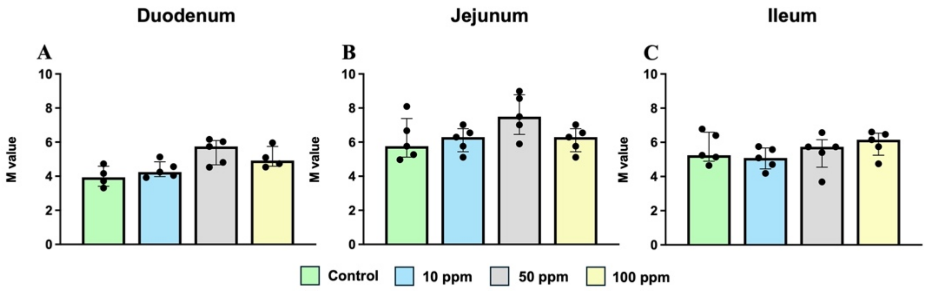

Supplementary Figure 4. Graphical representation of the absorption surface (M value) value across different segments of the small intestine. Panels show data for (**A**) duodenum, (**B**) jejunum, and (**C**) ileum. Bars represent median ± IQR. Black circles correspond to individual animal values within each group.

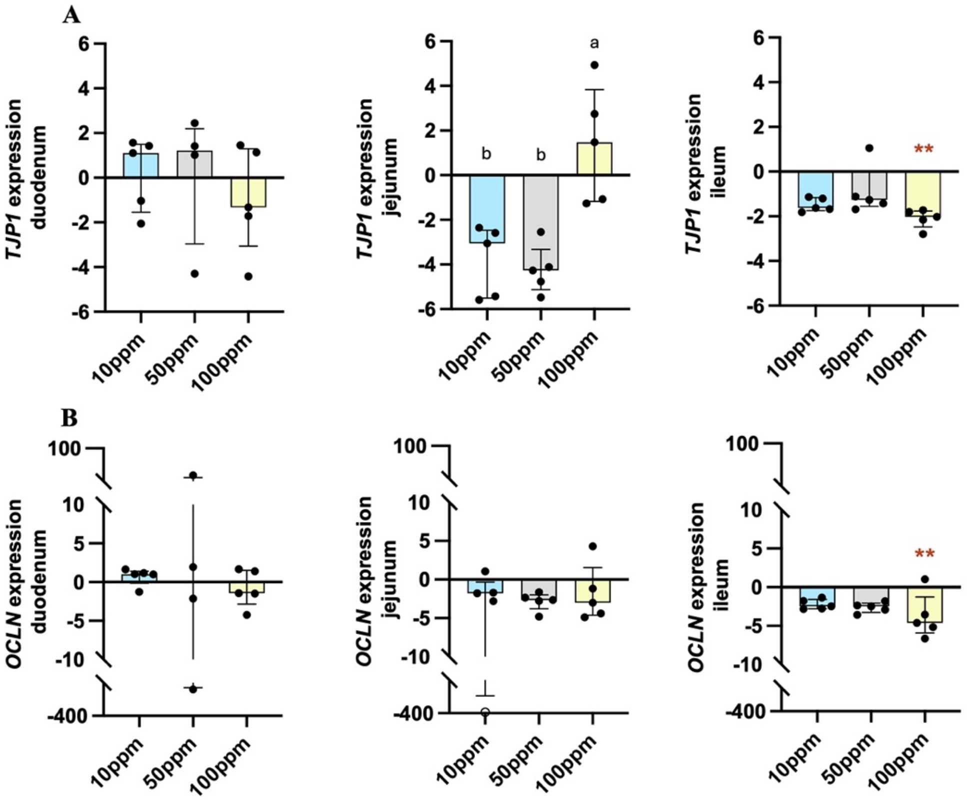

Supplementary Figure 5. Graphical representation of the mRNA expression levels of (**A**) *TJP1* and (**B**) *OCLN*. Expression levels were transformed as –1/fold change for values < 1, to visually distinguish downregulation (values < 0) from upregulation (values > 1). Bars represent median ± IQR. Black circles correspond to individual animal values within each group. Letters above the bars indicate statistically significant differences between treatment groups ($P < 0.05$), and red asterisks indicate statistically significant differences compared to the control group, indicated by $P < 0.01$ (**).

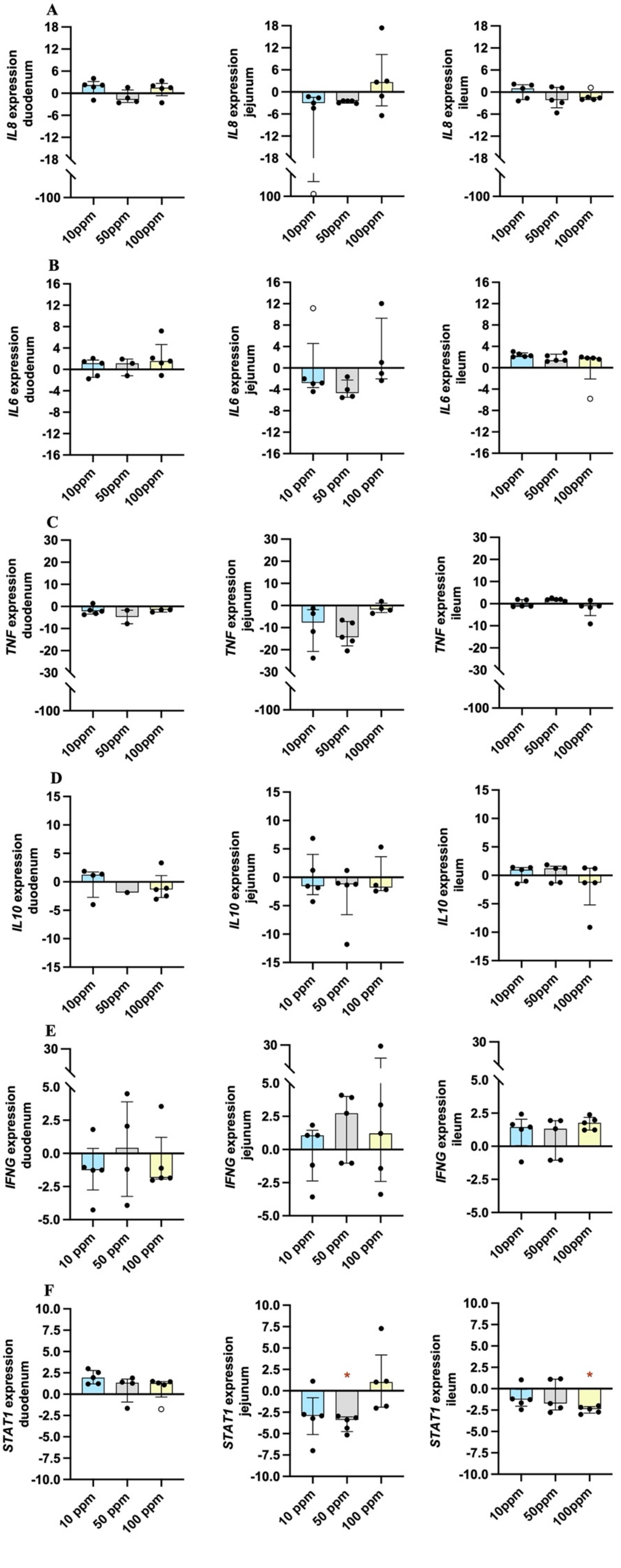
A
IL8 expression duodenum
IL8 expression jejunum
IL8 expression ileum
B
IL6 expression duodenum
IL6 expression jejunum
IL6 expression ileum
C
TNF expression duodenum
TNF expression jejunum
TNF expression ileum
D
IL10 expression duodenum
IL10 expression jejunum
IL10 expression ileum
E
IFNG expression duodenum
IFNG expression jejunum
IFNG expression ileum
F
STAT1 expression duodenum
STAT1 expression jejunum
STAT1 expression ileum
10ppm
50ppm
100ppm
10 ppm
50 ppm
100 ppm

Supplementary Figure 6. Graphical representation of the mRNA expression levels of (**A**) *IL8*, (**B**) *IL6*, (**C**) *TNF*, (**D**) *IL10*, (**E**) *IFNG*, and (**F**) *STAT1*. Expression levels were transformed as –1/fold change for values < 1, to visually distinguish downregulation (values < 0) from upregulation (values > 1). Bars represent median ± IQR. Black circles correspond to individual animal values within each group, and empty symbols represent outliers. Red asterisks indicate statistically significant differences compared to the control group, indicated by $P < 0.05$ (*), and $P < 0.01$ (**).

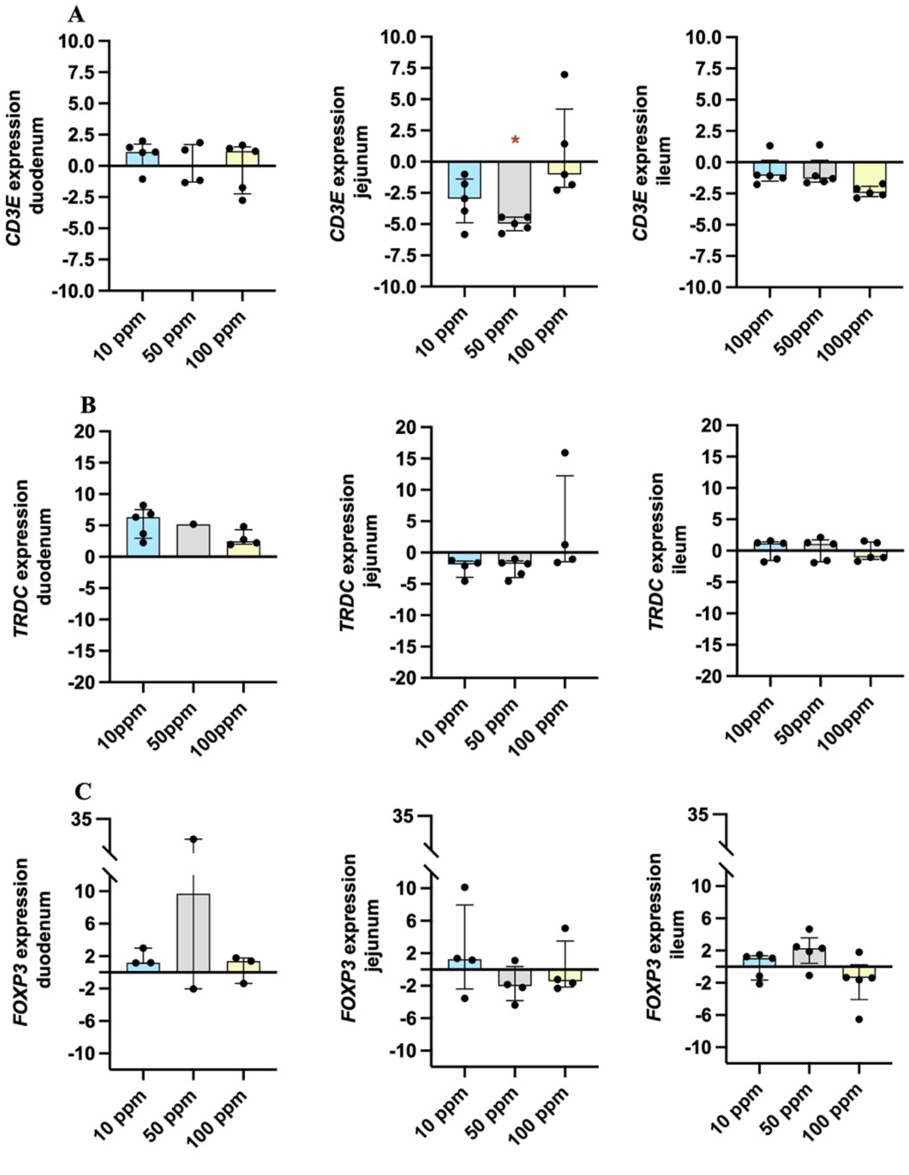

Supplementary Figure 7. Graphical representation of the mRNA expression levels of (**A**) *CD3*, (**B**) *TRDC*, and (**C**) *FOXP3*. Expression levels were transformed as –1/fold change for values < 1, to visually distinguish downregulation (values < 0) from upregulation (values > 1). Bars represent median ± IQR. Black circles correspond to individual animal values within each group. Red asterisks indicate statistically significant differences compared to the control group, indicated by $P < 0.05$ (*).
